# Whole transcriptome sequencing of *Pseudomonas syringae* pv. *actinidiae-*infected kiwifruit plants reveals species-specific interaction between long non-coding RNA and coding genes

**DOI:** 10.1038/s41598-017-05377-y

**Published:** 2017-07-07

**Authors:** Zupeng Wang, Yifei Liu, Li Li, Dawei Li, Qiong Zhang, Yangtao Guo, Shuaibin Wang, Caihong Zhong, Hongwen Huang

**Affiliations:** 1 Key Laboratory of Plant Resources Conservation and Sustainable Utilization, South China Botanical Garden, the Chinese Academy of Sciences, Guangzhou, Guangdong 510650 China; 2 Guangdong Provincial Key Laboratory of Applied Botany, Guangzhou, Guangdong 510650 China; 3 University of Chinese Academy of Sciences, Beijing, 100049 China; 4 Key Laboratory of Plant Germplasm Enhancement and Specially Agriculture, Wuhan Botanical Garden, the Chinese Academy of Sciences, Wuhan, Hubei 430074 China

## Abstract

An outbreak of kiwifruit bacterial canker disease caused by *Pseudomonas syringae* pv. *actinidiae* (Psa) beginning in 2008 caused disaster to the kiwifruit industry. However the mechanisms of interaction between kiwifruit and Psa are unknown. Long noncoding RNAs (lncRNAs) are known to regulate many biological processes, but comprehensive repertoires of kiwifruit lncRNAs and their effects on the interaction between kiwifruit and Psa are unknown. Here, based on in-depth transcriptomic analysis of four kiwifruit materials at three stages of infection with Psa, we identified 14,845 transcripts from 12,280 loci as putative lncRNAs. Hierarchical clustering analysis of differentially-expressed transcripts reveals that both protein-coding and lncRNA transcripts are expressed species-specifically. Comparing differentially-expressed transcripts from different species, variations in pattern-triggered immunity (PTI) were the main causes of species-specific responses to infection by Psa. Using weighted gene co-expression network analysis, we identified species-specific expressed key lncRNAs which were closely related to plant immune response and signal transduction. Our results illustrate that different kiwifruit species employ multiple different plant immunity layers to fight against Psa infection, which causes distinct responses. We also discovered that lncRNAs might affect kiwifruit responses to Psa infection, indicating that both protein-coding regions and noncoding regions can affect kiwifruit response to Psa infection.

## Introduction

Kiwifruit is becoming an increasingly popular fruit worldwide owing to its high vitamin C content and balanced nutritional components of minerals, dietary fiber and health-promoting metabolites^1–3^. So far commercial kiwifruit plantings have reached more than 228,778 hectares with an annual production of 3 million tons worldwide (http://faostat.fao.org). However, since bacterial canker disease caused by a highly virulent strain of *Pseudomonas syringae* pv. *actinidiae* (Psa) was first reported in Italy in 2008, and subsequently found in other producing countries, the world kiwifruit industry has suffered a devastating blow^4^. Symptoms of this disease are characteristic dark brown spots surrounded by yellow haloes on leaves, and cankers with copious reddish exudate production on twigs and stem^4^. Psa has caused severe decline of production and death of affected kiwifruit vines even loss of entire commercial orchard^5^. Unfortunately, no effective measures has been employed to mitigate this disaster.

Psa is a hemibiotrophic pathogen of the *P*. *syringae* complex, which contains a large variety of plant pathogens, leading to diverse diseases of both wild and crop plants^6^. On the basis of geographic origin, physiological and biochemical characteristics, and also genomic evidence, Psa can be divided into five separate clades (now popularly referred as biovars 1–5)^7–9^. Biovar 1 comprises strains from the Japanese and Italian outbreaks of 1984 and 1992 respectively^8^. This biovar contains an argK-tox gene cluster, which regulates synthesis of phaseolotoxin and hydrolyzes it into the compound PSOrn which inhibits ornithine carbamoyl transferase (OCTase)^10^. OCTase is involved in the urea cycle of both prokaryotes and eukaryotes, and inhibition of OCTase leads to the formation of chlorotic haloes on leaves of the host^11^. Biovar 2 is represented by strains isolated in South Korea during the epidemics of the 1990s and it can produce coronatine^12^, while biovar 4 was isolated from New Zealand and Australia and has low virulence without significant symptoms. Recently, the biovar 4 has been identified as *de novo* pathovar *P*. *syringae* pv. *actinidifoliorum* (Pfm) on the basis of pathogenic and phylogenetic differences^13^, and biovar 5 is currently only found in a localized area of Japan^9^. It is notable that biovar 3 is responsible for the current epidemic of kiwifruit canker which isolated from Europe, New Zealand, Chile and Asia^14^. Strains of biovar 3 cannot produce both phaseolotoxin and coronatine, but possess four putative clade-specific hop genes: hopH1, hopZ5, hopAM1-2, and hopAA1-2, which encode effector proteins as part of the type III secretion system in *P*. *syringae* species^7^. In kiwifruit, these Psa strains can enter the host through natural orifices or wounds, and can also be disseminated through pollen^15^. However increasing investigations indicate that leaf colonization is one of the most important phases of the pathogen cycle of disease, because Psa can migrate systemically within the leaf veins after stomatal penetration^16^.

When pathogenic microbes arrive on the cell surface of a host plant, microbe-derived conserved molecules are detected and the innate immune system of the host is triggered. As microbe-associated molecular patterns (MAMPs), these molecules are recognized by the plant membrane proteins recognition receptors (PRRs)^17^, which result in the invasion of pathogenic microbes is suppressed. This immune system is known as pattern-triggered immunity (PTI)^18^. In turn, the pathogenic microbes secrete effectors which overcome PTI by interfering with MAMP detection and/or subsequent defense signaling. On the other side, the plants coevolve resistance (R) genes inhibiting the effect of effectors secreted by pathogenic microbes. This layer of the plant immune system is effector-triggered immunity (ETI)^17, 18^. Comparatively, PTI is a relatively ancient and broad-spectrum form of immunity, while ETI is more evolved and exhibits a species- or clade-specific pattern.

Numerous genes are involved in the molecular interactions of the plant immune system. In kiwifruit, 96 putative nucleotide-binding sites and leucine-rich repeat (NBS–LRR) genes related to ETI, and 261 putative PRR genes associated with PTI, have been identified by comparative genomic analysis^2^. Plant hormones also play an important role in plant immunity^17, 18^. Transgenic tobacco and *Arabidopsis* expressing salicylate hydroxylase, which interferes with accumulation of salicylic acid (SA), cannot induce systemic acquired resistance, leading to increased susceptibility to viral, fungal and bacterial pathogens^19^. Similarly, DELLA proteins can affect plant immune responses by regulating accumulation of jasmonic acid (JA) and SA^20^, and the *Pseudomonas* type III effector HopQ1 can suppress accumulation of the flagellin receptor FLS2 by activation of cytokinin signaling^21^. Transcription factors (TFs) can also participate in plant immune responses to pathogens. WRKY is a large TF family which can regulate plant responses to diverse biotic and abiotic stresses^22^. ERF subfamily members participate in the regulation of genes responsive to biotic stress, in particular to genes in relation to JA and ethylene hormone signaling pathways^23^. The TGA-bZIP family can also regulate the plant defense system through the SA signaling pathway^23^.

The role of plant noncoding RNAs, in particular the major type of long noncoding RNAs (lncRNAs) has not been thoroughly investigated. LncRNAs have been defined as noncoding RNAs more than 200 base pairs (bp) in length^24^, and can be classified into four types including natural antisense lncRNAs, intronic lncRNAs, overlapping lncRNAs (overlapping with protein-coding genes) and intergenic lncRNAs on the basis of their genomic positions^25^. According to genome-wide analysis, lncRNAs are widespread and present in both plants and animals, including human^26^, mouse^27^, *Arabidopsis thaliana* ^28^, *Oryza sativa* ^29^, *Zea mays* ^30^ and *Gossypium spp*.^31^. The functions of lncRNAs can be associated with human cancer cell development, plant photomorphogenesis, abiotic and biotic stress responses, and other diverse biological processes^32–34^. In rice, the LDMAR (long-day-specific male-fertility-associated RNA) has been verified as an lncRNA which regulates photoperiod-sensitive male sterility^35^. Meanwhile in *Arabidopsis*, an intronic lncRNA COLDAIR (cold assisted intronic noncoding RNA) was found to be a required component for vernalization-mediated epigenetic repression of the flowering locus FLC^36^. A recent study showed that kiwifruit lncRNAs were related to fruit development and ripening^37^. However, the systematic identification and characterization of lncRNAs in plants is relatively limited, in particular for fruit crops and non-model organisms. The role of lncRNAs in relation to plant host–pathogen interaction is also unclear.

It is critically important to understand species-specific host–pathogen responses, which facilitate designing strategies and/or generating germplasm to rescue the kiwifruit industry in future. Due to the devastating effect of Psa on kiwifruit mainly derived from *Actinidia chinensis* (Ac) cultivars, the molecular patterns in relation to Psa resistance/susceptibility of other *Actinidia* taxa was never investigated. We chose *A*. *arguta* (Aa), *A*. *eriantha* (Ae) and Ac as representative taxa to investigate the differences in the molecular interactions between host and Psa. The phylogenetic distinction between Aa and Ac is greater than that between Ae and Ac^38^. We also included two cultivars with different ploidy levels ‘Hongyang’ (AH: diploid) and ‘Jinyan’ (AJ: tetraploid) as intraspecific samples of Ac. In the Ac species complex, both cultivars ‘Hongyang’ and ‘Jinyan’ are developed from the variety *A*. *chinensis* var. *chinensis*. Cultivars derived from the variety *A*. *deliciosa* are also widely planted. However, *A*. *deliciosa* is quite closely related to the two cultivars (AH and AJ) originated from Ac^39^ and the consideration of cost for RNA-sequencing, we excluded this variety in our analysis. The in-depth RNA sequencing of these materials at three stages of infection after Psa inoculation were carried out. We found that the expression of genes in different *Actinidia* taxa were remodeled in a species-specific pattern, corresponding to leaf symptoms associated with Psa infection. LncRNAs related to kiwifruit responses to Psa infection were also species-specifically co-expressed with protein coding genes in leaf tissues. The results indicate that both protein-coding and noncoding genomic regions can significantly correlate with plant resistance/susceptibility in the face of pathogenic invasion.

## Results

### Symptoms of Psa-inoculated kiwifruit species

**Figure 1:**
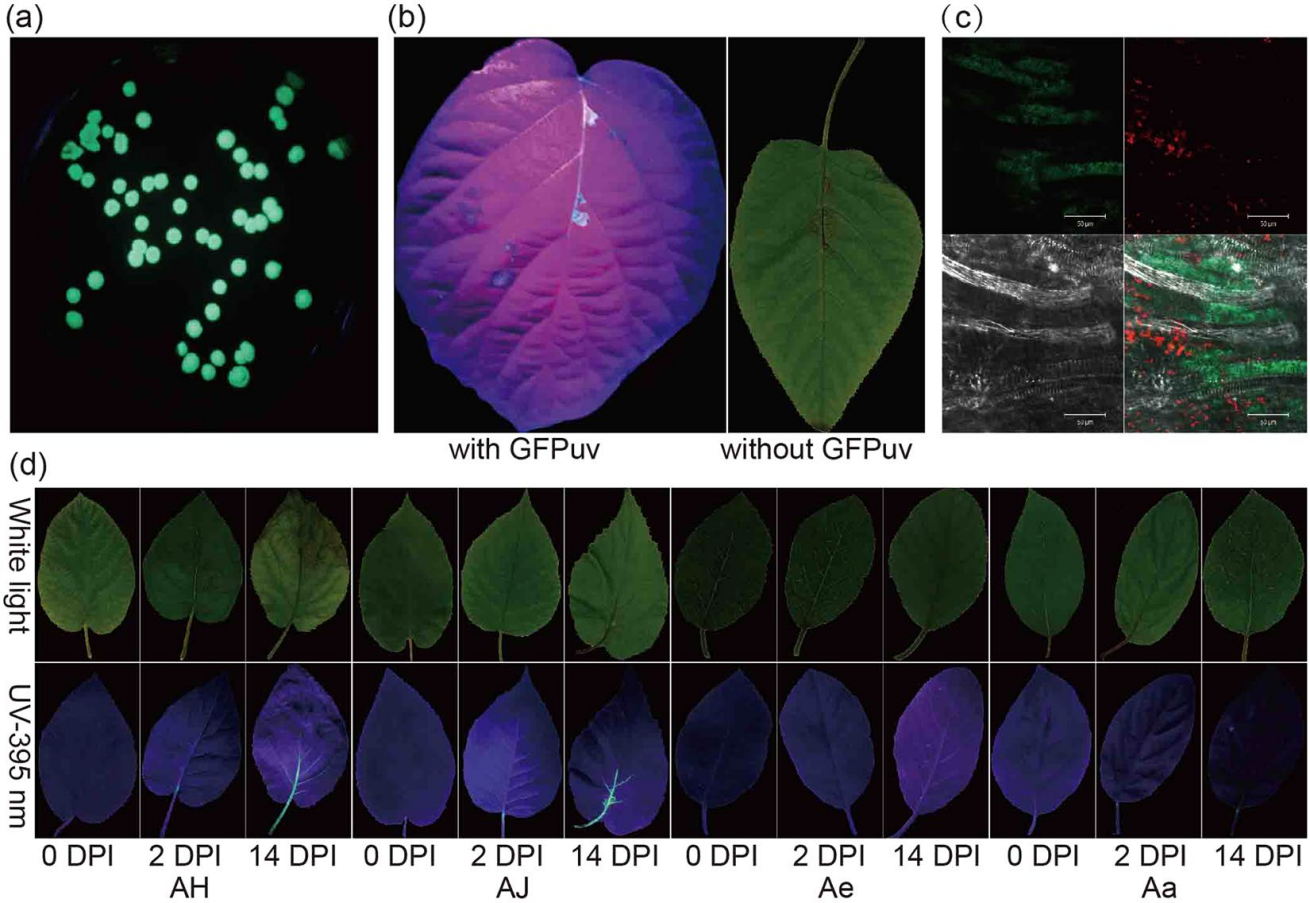
Visualization of GFPuv-labeled Psa and leaf symptoms of kiwifruit infected with transformed Psa. (**a**) Psa strain C48 transformed with GFPuv. (**b**) Leaf symptoms of Hongyang (AH) with invasion of C48 showing no effect on the toxicity of GFPuv-transformed Psa stain C48. (**c**) An AH leaf collected at 14 days post inoculation (DPI) examined under a Leica confocal laser scanning microscope with excitation at 488 nm and emitted light collected from 500 to 600 nm. Images were captured of green fluorescence (upper left), autofluorescence (upper right), natural light (low left) and combined image (low right). (**d**) Leaf symptoms of kiwifruit samples during Psa infection. Photographs were taken under natural light (upper panels) and UV light (395 nm, low panels).

Kiwifruit seedlings inoculated with C48, a highly-virulent Psa strain of biovar 3, were inspected to identify canker disease symptoms. We used a GFPuv-transformed strain to visualize the movement of Psa within kiwifruit leaf tissues. GFPuv generates 45-fold brighter green fluorescence and has lower toxicity to bacteria in comparison to the wild-type GFP. Its expression in transformed Psa was stable and fast on King’s B medium (KB) plates (Fig. 1a). No effects on the virulence of the GFPuv-expressing Psa was detected, since it produced the same symptoms on AH leaves as those inoculated by untransformed strains (Fig. 1b). The presence of GFPuv-expressing Psa within kiwifruit leaves was also clear and distinct when examined under a confocal microscope (Fig. 1c).

Leaf phenotypes were recorded at three stages of infection: day 0 (without inoculation), day 2 and day 14 post inoculation (DPI) for the four *Actinidia* materials investigated (Supplementary Table S1). Our results showed differences in the appearance of Psa symptoms on the leaves of *Actinidia* species at both 2 and 14 DPI (Fig. 1d). For the two samples of Ac (AH and AJ), we found typical leaf symptoms were present at 14 DPI, which were similar to those observed in Psa-infected orchards. Of the two cultivars, AH showed more severe leaf damage and shrinkage with the presence of necrotic lesions compared to AJ, although both revealed strong green fluorescence along leaf veins at this time-point (Fig. 1d). At 2 DPI, neither AH nor AJ showed any obvious leaf symptoms although weak green fluorescence was present around the inoculation points and leaf veins. For Ae and Aa, no obvious leaf symptoms were observed at either 2 or 14 DPI, and the green fluorescence along leaf veins was also weak although it was detectable (Fig. 1d).

### Sequencing of the Psa-infected kiwifruit transcriptome and identification of lncRNAs

We performed whole-transcriptome sequencing (RNA-Seq) of 24 RNA libraries constructed from leaf tissues collected at three stages (0, 2, and 14 DPI) of four materials (AH, AJ, Ae and Aa) with biological replicates considered (*n* = 2 biological replicates per stage per material). A total of 2,304 million paired-end reads were yielded. After stringent filtering, clean reads were aligned to the kiwifruit genome reference (http://bioinfo.bti.cornell.edu/cgi-bin/kiwi/home.cgi) and Psa strain NZ13 genome reference, respectively^2, 7^. In mapping to the kiwifruit reference, we found the highest mapping rate (86.51%) for AH (Supplementary Table S2), from which the reference was assembled. We found similar mapping rates for both AJ (84.80%) and Ae (84.12%), but a relatively low rate was detected for Aa (76.48%), consistent with the phylogenetic difference between these samples and species. We found that approximately 0.08–1.40% of reads mapped to the Psa reference, with variation due to differences in species and collection time (Supplementary Table S2).

For all mapped reads, we performed “*de novo*” transcriptome assembly, which resulted in retrieval of 214,688 transcripts. We used the Annocript pipeline^40^ and integrated the reference information to annotate these transcripts, resulting in annotation of 119,017 kiwifruit protein-coding transcripts which originated from 39,040 loci (Supplementary Data S1). Owing to the generation of multiple transcripts from the same gene through alternative splicing, 24,045 skipped exon (SE) and 891 mutually exclusive exon (MXE) alternative splicing (AS) events were obtained. Amongst these, 36 SE and 15 MXE splicing events were significantly different between samples (Supplementary Data S2, Supplementary Figure S1). The AS events were increased in AH, AJ and Ae, but slightly decreased in Aa during Psa infection, but the numbers of significant AS events were reduced in AH, AJ and Ae, but increased in Aa (Supplementary Figure S1). We found that several genes involved in significant AS events were related to plant defense responses (Supplementary Data S2), such as *Achn060421* which encoded glutamine amidotransferase subunit PDX 2 regulating the biosynthesis of vitamin B6, and were responsible for plant defense responses^41^.

**Figure 2:**
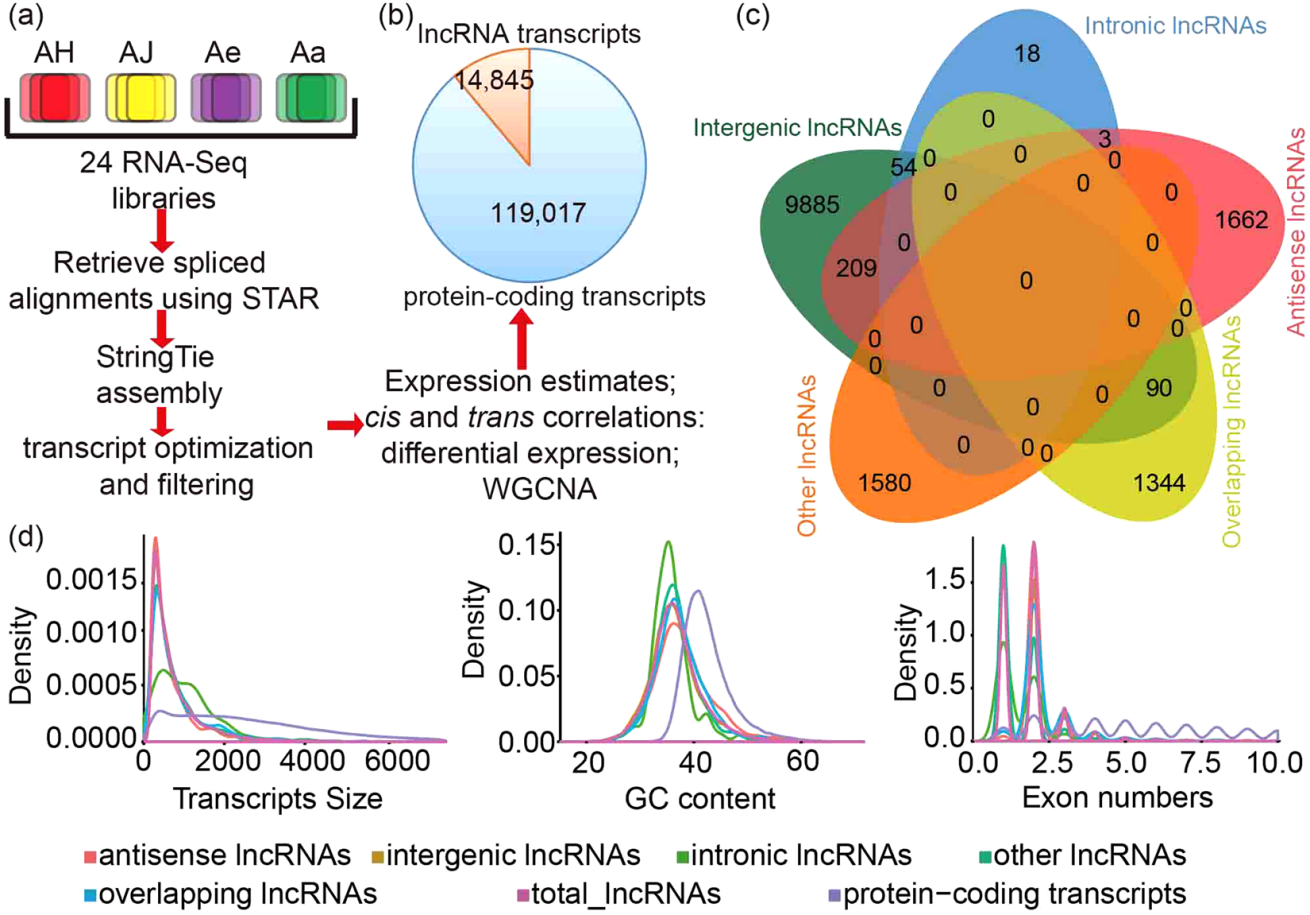
Overview of kiwifruit transcriptomes and characteristics of lncRNAs. (**a**) Samples and pipeline for transcriptomic analyses. (**b**) Total number of protein-coding transcripts and lncRNAs. (**c**) Venn distribution of different lncRNA types. (**d**) Density distribution of transcript length, GC content and exon number for both protein-coding and lncRNA transcripts.

The lncRNAs were identified using an ‘*ab initio*’ assembly pipeline (Figs 2a and S2a). We filtered annotated transcripts, and excluded those less than 200 bp long or with an open reading frame (ORF) greater than 100 amino acids long. The comprehensive annotations comprised a total of 14,845 lncRNA transcripts (Fig. 2b), of which 68.97% (10,238) were intergenic lncRNAs, while 12.63% (1,874), 0.51% (75), 9.66% (1434) and 10.6% (1,805) were antisense lncRNAs (NATs), intronic lncRNAs, overlapping lncRNAs and other lncRNAs, respectively (Fig. 2c). All these lncRNAs were substantially expressed at an FPKM (‘fragments per kilobase of exon per million reads mapped’) value greater than 0 in at least one sample although most lncRNAs were expressed at a low level (Supplementary Figure S2b). The mean length of lncRNAs was 774 bp with an average GC content of 36.78%, both of which were less than those of protein-coding genes (~2.8 kb and 42.49%, respectively) (Fig. 2d). Most lncRNA transcripts (88.07%) contained 1–2 exons, but the exon number also significantly varied between types, with 50.93% of intergenic lncRNA and 42.67% of intronic lncRNA containing less than or equal to 2 exons, while most overlapping lncRNAs (94.98%) and antisense lncRNAs (98.03%) harbored relatively more exons (exon numbers ≥2) (Fig. 2d). Our results thus revealed divergent features of lncRNAs comparing with those of protein-coding transcripts, consistent with previous reports^28–31^.

For all kiwifruit transcripts, 74% of protein-coding genes and 72% of lncRNAs were expressed (FPKM >1) in both replicates of at least one stage per genotype, indicating a strong correlation of gene expression between replicates (mean correlation coefficient = 0.92, s.d. = 0.03, Supplementary Figure S3a). To verify the RNA-seq expression data, we performed quantitative PCR (qPCR) analysis of 51 genes (33 protein-coding genes and 18 lncRNA genes, Supplementary Table S5) in all samples with biological replicates. High concordance between the qPCR expression results and the RNA-seq data were verified (mean correlation coefficient = 0.88, s.d. = 0.05, Supplementary Table S5 and Supplementary Figure S3b).

### Species-specific gene expression profiling during Psa infection

**Figure 3:**
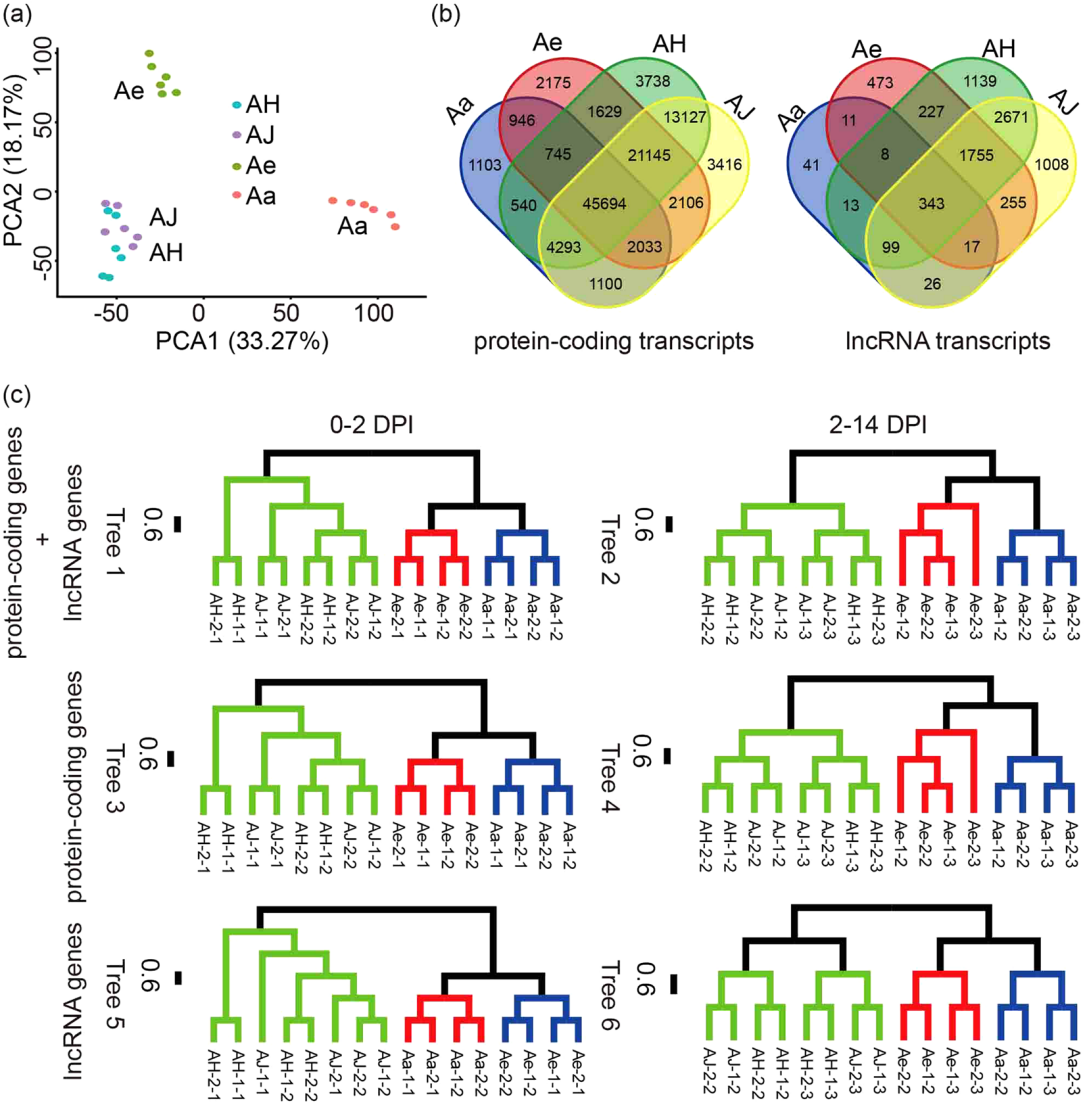
Species-specific expression patterns of differentially-expressed transcripts. (**a**) PCA clustering of both protein-coding and lncRNA transcripts. (**b**) Venn distribution of the respective protein-coding and lncRNA transcripts for each *Actinidia* material studied. (**c**) Hierarchical clustering of differentially-expressed transcripts for both protein-coding and lncRNA transcripts. The green, red and blue clades represent samples from the species Ac, Ae and Aa respectively.

The overall expression pattern of lncRNA and protein-coding genes of *Actinidia* taxa were characterized. Principal component analysis (PCA) based on dataset of both types of genes, revealed three distinct groups in relation to the three species investigated (Fig. 3a). Comparatively, lncRNA genes were expressed two times more in species-specific manner than protein-coding genes during Psa infection, with 17.9% of lncRNA transcripts and only 8.7% of protein-coding genes was expressed in one genotype (Fig. 3b). Furthermore, significant difference were detected on gene expression with Psa infection. At 0 DPI, the expression profiles of all samples were diverse and random, but at 2 DPI there was reduced expression of protein-coding transcripts (11.6% vs 14.8%) although the lncRNAs expressed were similarly diverse (1.3% vs 1.2%) (Supplementary Table S3). At 14 DPI with top infection of Psa within leaf tissues, only 6.3% of protein-coding transcripts and 0.9% of lncRNAs were expressed differently, suggesting a more focused response of gene expression in relation to Psa infection in kiwifruit, despite minor differences being observed between species (Supplementary Table S3).

We constructed sample trees based on gene expression matrix of the combined tree (Tree 1 and 2, Fig. 3c), the protein-coding genes (Tree 3 and Tree 4) and lncRNA genes (Tree 5 and Tree 6). Genes in these sets were expressed differentially in at least one pairwise comparison during the 0–2 DPI and the 2–14 DPI stages. There is no significantly affected on tree topologies according to the different gene datasets and sample collecting stages. However consistent with the PCA, the grouping patterns were more dominated by species examined *per se* (Fig. 3c). Within each species, samples tended to cluster together in accord with collecting stages (Fig. 3c). Some clustering patterns were mixed at the 0 DPI stage (e.g., AH and AJ samples on Tree1, 3 and 5), which could possibly reflect random gene expressions of kiwifruit leaves without Psa infection as mentioned above.

For differentially-expressed protein-coding genes, the gene ontology (GO) and Kyoto Encyclopedia of Genes and Genomes (KEGG) pathway function enrichments were surveyed. We found the differentially-expressed transcripts were significantly related to the biological processes of metabolism, response to stimuli, immune system and cellular processes (Supplementary Figure S4). The transcripts within each GO term were varied dramatically between *Actinidia* taxa, with the highest diversity found in both AH and AJ of Ac (Supplementary Figure S4). Moreover, an overall heterogeneous landscape of KEGG pathway enrichments between taxa was further identified (Supplementary Figure S5), in which the up- or down-regulated genes during the 0–2 and 2–14 stages were vastly different (Supplementary Table S4). For example, transcripts within the photosynthesis pathway were severely repressed in both Aa and Ac (AH and AJ), but highly expressed in Ae (Supplementary Figure S6a). Fatty acid metabolism-related genes were up-regulated in Aa, but diversely regulated in both Ae and Ac (Supplementary Figure S6b). We also detected species-specific patterns of gene expression in energy metabolism, carbohydrate metabolism, amino acid metabolism and biosynthesis of secondary metabolites (Supplementary Figure S4). These biological processes are all involved in the complex energy generation and nutritional assignment requirements of the host, contributing to both growth and survival of kiwifruit plants in the face of Psa infection.

For all differentially-expressed transcripts, the protein domain enrichment was analyzed. Enriched protein domains/families have showed to be associated with HSP90 family proteins, serine/threonine protein kinase catalytic domains (STKc), P kinase, catalytic domain of protein kinases (PKc), and leucine-rich repeats (LRR). These conserved domains/families are closely related to the plant immune system and immune responses (*q*-value < 0.05), consistent with the results of KEGG pathway enrichment analysis (Supplementary Data S3).

### Differences in gene expression in relation to plant immunity

To identify genes associated with plant immune-related pathways, we investigated the FPKM value of each differentially-expressed transcript. We found changes in gene expression in both PTI and ETI layers. To verify the potential functions of plant immune-related genes annotated, we constructed phylogenetic trees with genes of four representative gene families (cyclic nucleotide gated channels/calcium-dependent protein kinase/*MAPK*/*WRKY*) in both kiwifruit and other species. It illustrated that the grouping patterns was similar in both kiwifruit and other species (Supplementary Figure S7). Taking the CPKs gene family as an example, four distinct subgroups were identified and genes were preferentially clustered on the basis of their phylogenetic relationships rather than species, which were consistent with previous research, suggesting potentially similar functions and immune pathways of these genes involved between kiwifruit and other species.

**Figure 4:**
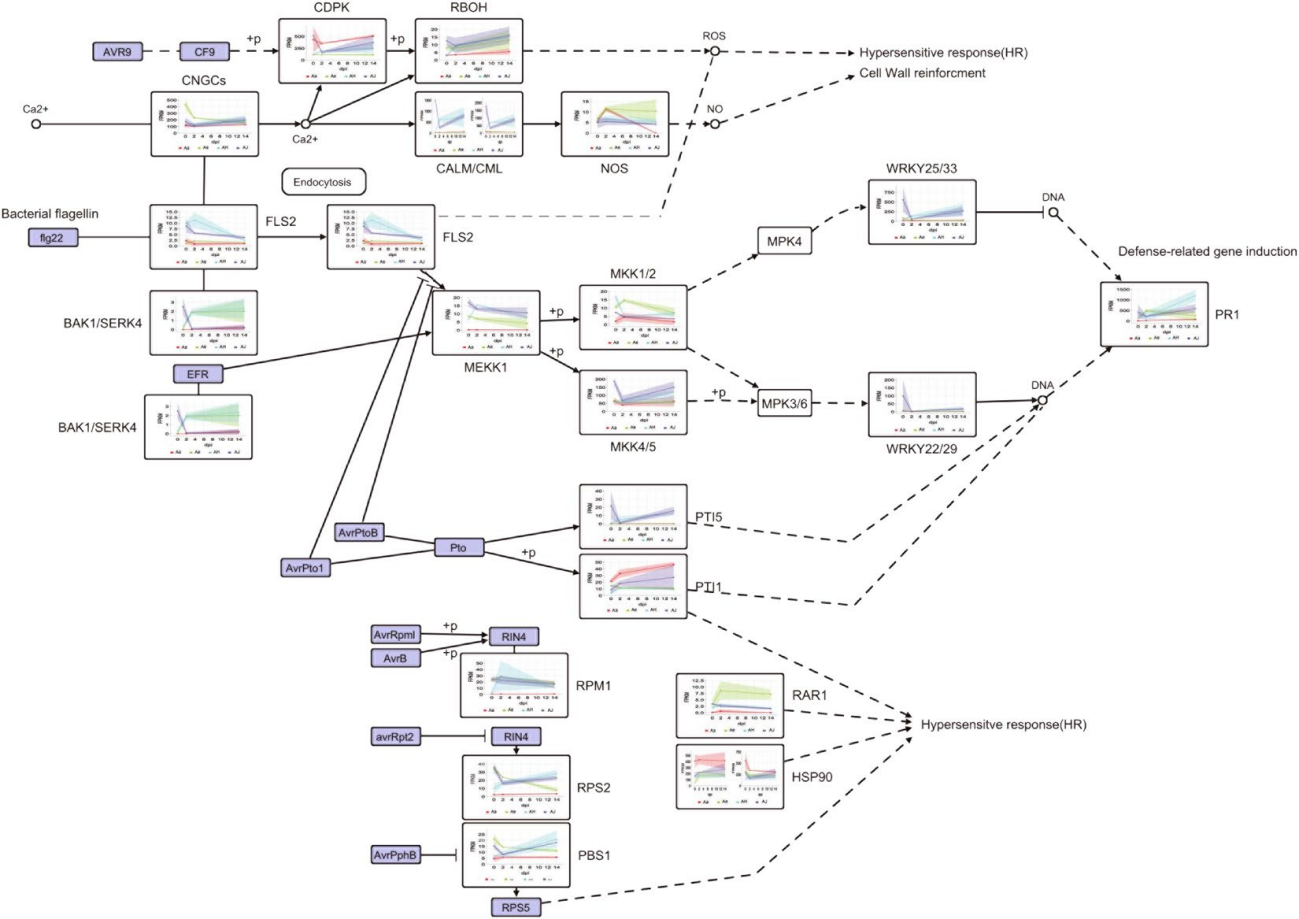
Visualization of plant–pathogen interaction pathways. Changes in gene expression are shown in line graphs. Each line represents the changes of a sample across three consecutive sampling time-points. The red, green, cyan and blue lines represent AH, AJ, Aa and Ae respectively.

Under the hypothesis of functional similarity for phylogenetically close genes in both kiwifruit and Arabidopsis, we further inferred the potential roles of genes played in the pathways at both PTI and ETI layers. Two genes, *Achn376371* (a *FLS2*-like gene) and *Achn109861* (an *EFR*-like gene) were possibly activated by Psa-associated MAMPs (Fig. 4), triggering the MAPK signaling cassette which was involved in activating defense genes to produce antimicrobial compounds in kiwifruit^41^. Both FLS2 and EFR are LRR receptor-like serine/threonine-protein kinase in *Arabidopsis* ^42^. This was further supported by changes in the expression of both genes *Achn180781* and *Achn247141* (mitogen-activated protein kinase kinase kinase 1, a *MEKK1P*-like gene). In *Arabidopsis*, MEKK1P activated by FLS2 or EFR, which were phosphorylated into MKK1/2 (mitogen-activated protein kinase kinase 1) and MKK4/5 (mitogen-activated protein kinase kinase 4/5) respectively^43^. Correspondingly, changes in the expression of a *MKK1/2*-like gene (*Achn156001*) and *MKK4/5*-like gene (*Achn301271* and *Achn164271*) were observed in kiwifruit (Fig. 4). Furthermore, the MAPK signaling cassette can activate transcription factors WRKY25/33 and WRKY22/29, affecting expression of defense-related genes such as PR1 (pathogenesis-related protein 1) in Arabidopsis, while similar expression variations on a *WRKY25/33*-like gene (*Achn314301*), *WRKY22/29*-like genes (*Achn245731* and *Achn278571*) and a *PR1*-like gene (*Achn146991*) were found in kiwifruit (Fig. 4). During Psa infection, some genes in the *CNGCs* (cyclic nucleotide gated channels) family were up-regulated to increase the concentration of calcium cation possibly (Fig. 4), and then affected expression of some genes in *CDPK* (calcium-dependent protein kinase) family, *RBOH* (respiratory burst oxidase) family, and calmodulin family, leading to regulation of the production of reactive oxygen species (ROS) and programmed cell death/hypersensitive response.

We clustered all samples based on the gene expression of differentially-expressed transcripts within plant-pathogen interaction pathways. The topological structure of the sample tree was identified according to leaf symptoms at 14 DPI with infection of Psa (Supplementary Figure S8). Samples of AH and AJ which exhibited severe symptoms were classified in the same clade, while samples of Ae and Aa without obvious symptoms were clustered into another clade (Supplementary Figure S8) indicating that leaf symptoms were in accord with the expression level of genes in the plant–pathogen interaction pathway., Based on gene expression of transcripts within the plant–pathogen interaction pathway, we identified 25 (originating from 18 genes), 13 (originating from 10 genes) and 4 transcripts (originating from 4 genes) which were uniquely expressed in Ac, Ae and Aa, respectively (Supplementary Figure S9, Supplementary Data S4). For example, many transcripts annotated as CDPK/RBOH and CaM/CML/NOA1 were expressed in both Ac and Aa, but only transcripts annotated as CDPK were found in Ae (Supplementary Figure S9). Both CDPK/RBOH and CaM/CML/NOA 1 are important regulators in plant PTI and can induce bursts of ROS and NO respectively, leading to diverse effects in plant–pathogen interactions^44^. This result indicated that there is a significant difference between different kiwifruit species in relation to the PTI defense presented.

### Correlation of coding gene expression and lncRNA gene expression

**Figure 5:**
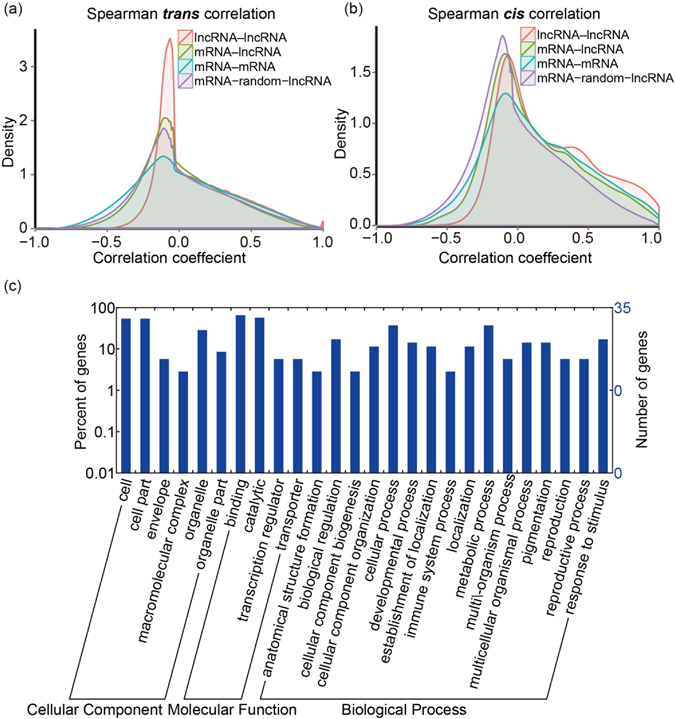
The *trans* and *cis* correlation analysis for gene pairs. (**a**) Density distribution of *trans* correlations. The protein-coding–protein-coding gene pairs, lncRNA–protein-coding pairs and lncRNA–lncRNA pairs were each calculated separately. (**b**) Density distribution of *cis* correlations. (**c**) GO term distribution of protein-coding genes strongly correlated to lncRNAs (*r*_s_ > 0.8).

To investigate the correlation of gene expressions within and between protein-coding genes and lncRNA genes, we analyzed pairwise expression correlations between genes across all samples. The *trans* correlations of expression was analyzed using a definition of a pair of genes separated by a distance of >2 megabases, or located on different chromosomes. We found that the expression of both protein-coding genes and lncRNA genes tended to be more positively correlated than negatively correlated with each other in *trans*, in which 1.33% of protein-coding gene–protein-coding gene pairs, 0.79% of protein-coding gene–lncRNA gene pairs, and 1.94% of lncRNA gene–lncRNA gene pairs had a Spearman correlation coefficient (|rho|) (*r*_s_) of >0.8, versus 0.10%, 0.01%, and 0.00%, respectively, with an *r*_s_ of <−0.8, of a total of 1.58 × 10^9^ *trans* correlations tested (Supplementary Data S5). We observed a tendency for greater positive correlation within lncRNA–lncRNA gene pairs than between other pairs (Supplementary Data S5). For lncRNA gene–lncRNA gene pairs, the bias toward positive correlations was greater than that obtained for a control set of *trans* correlations. The expression of protein-coding genes and lncRNA genes in control correlation was randomly ‘shuffled’, but the correlations for protein-coding gene–protein-coding gene pairs and protein-coding gene–lncRNA gene pairs were lower than that of control (Fig. 5a). This indicates that *trans*-regulatory effects between lncRNA genes and protein-coding genes are not obvious in kiwifruit during Psa infection.

The *cis* correlations of expression was analyzed among pairs of genes both located within a genomic window of 10 kb. A much higher proportion of positive correlations was estimated in 1.69 × 10^5^ *cis* correlations, and no negative correlations with an *r*_s_ of <−0.8 was detected in lncRNA gene–lncRNA gene pairs (Supplementary Data S5). We further found a higher proportion of positive correlations among *cis* correlations than among *trans* correlations, for both protein-coding gene–protein coding gene pairs and protein-coding gene–lncRNA gene pairs (Supplementary Data S5). In all cases, the number of positive *cis* correlations was slightly higher than those obtained for a control set, but this was not true for negative *cis* correlations (Fig. 5b).

GO analysis of protein-coding genes with strong positive *cis* correlations (*r*_s_ > 0.8) with lncRNA genes revealed significant enrichment for genes encoding products involved in response to stimulus and immune system process, metabolic processes (Fig. 5c), suggesting the roles played by lncRNAs in relation to kiwifruit host responses during Psa infection.

### Co-expression modules of lncRNAs and coding genes

**Figure 6:**
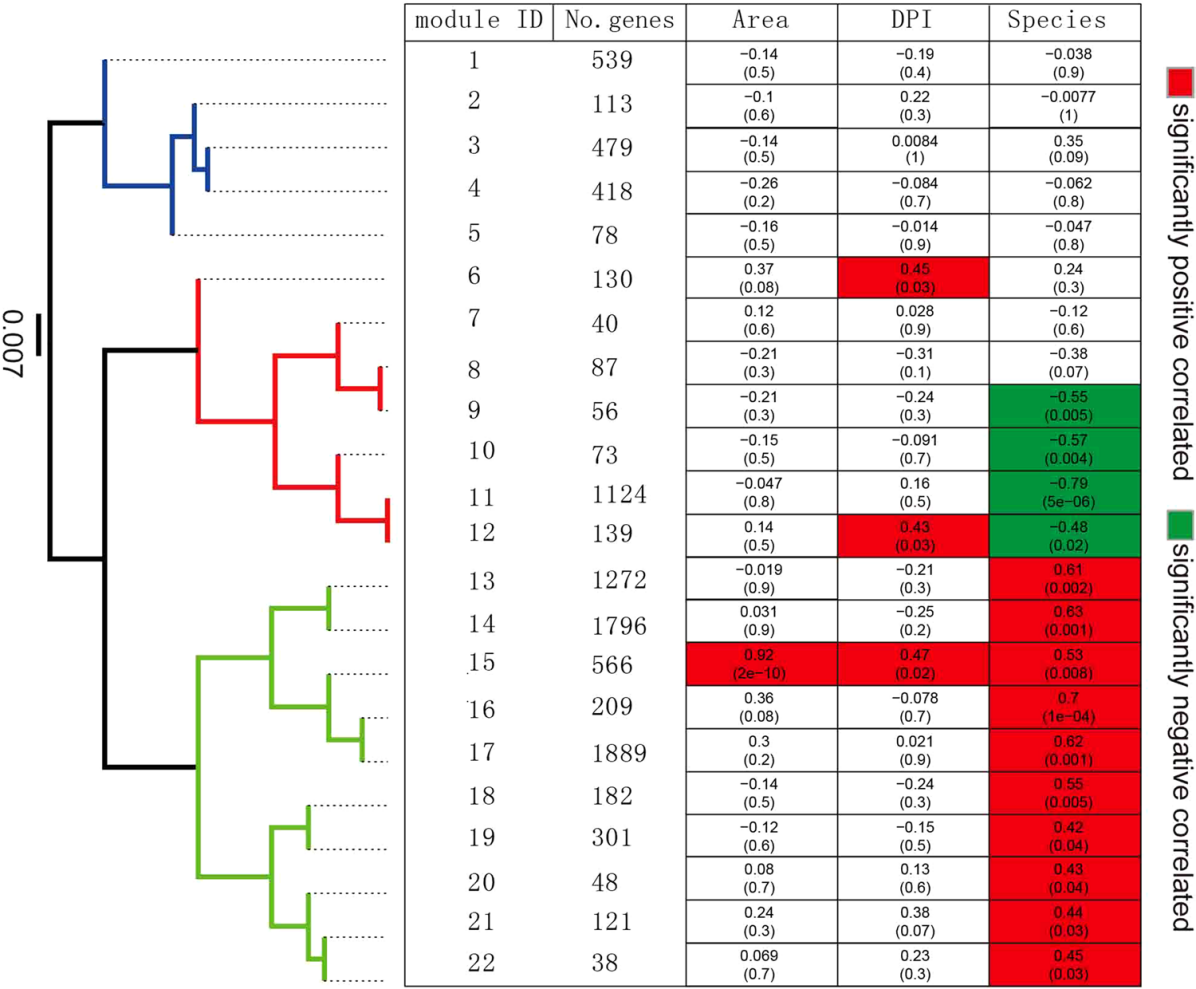
Correlation between co-expressed WGCNA module eigengenes and phenotypic traits (leaf area with green fluorescence, DPI and species). Modules were clustered based on eigengenes, resulting in three distinct clades, indicated by different colors. Both the correlation coefficients and the *P-*value (in brackets) are shown. Significant positive and negative relationships are highlighted in red and green color respectively.

To detect clusters of highly interconnected genes and explore the function of lncRNAs on the basis of protein-coding genes, we performed weighted correlation network analysis (WGCNA)^45^. WGCNA clusters genes with strong co-expression into modules, and the impact of each gene in a given module is calculated as a module membership which allows us to further pick out the hub genes (highly connected genes which contribute most to the module). The combined FPKM matrix of differential expression in both the lncRNA and protein-coding transcripts was analyzed in at least one pairwise comparison of all RNA-seq libraries. An expression matrix of 9,698 transcripts (including 998 lncRNAs and 8,700 protein-coding transcripts) were obtained for further analyzed (Supplementary Data S6)^45^. The final co-expression networks comprised 22 modules with an average number of 440 transcripts in each module (s.d. = 563.7) (Fig. 6).

We calculated the eigengene, which is a value for each sample for each module representative of the gene expression profiles of the samples in a module^46^. To investigate the relationships between the set of co-expressed genes (protein-coding genes and lncRNAs) and the external phenotypic traits, we correlated eigengenes of each module to the Psa-infected leaf area (determined using green fluorescence at 14 DPI), the three leaf tissue collecting stages (0, 2 and 14 DPI) and the three species phenotypes (Ac, Ae and Aa) (Fig. 6 and Supplementary Data S7). Expression of 15 out of 22 modules was significantly correlated with two of the three *Actinidia* species (Fig. 6). We further grouped modules on the basis of eigengenes, resulting in three distinct clades reflecting positive, negative and non-significant relationships between modules and traits of Ac, Aa and Ae, respectively.

There are three modules (modules 6, 12 and 15) which were strongly associated with the sampling stages of 14 DPI (Fig. 6). The module 15 was significantly related to both the Psa infected leaf area and the Ac species phenotype. This module was co-opted for all three phenotypic traits, suggesting the evolution of a set of linked and co-expressed genes particularly for Ac responsible, was accord with the most obvious and consistent leaf symptoms of both samples AH and AJ of this species after Psa inoculation (Fig. 1).

### Species-specific modules corresponding to kiwifruit defense responses to Psa infection

In 15 modules which exhibited significant positive and negative relationships to Ac and Aa phenotypes respectively, the biological relevance and functional significance in relation to species-specific resistance/susceptibility to Psa infection were investigated. Across all 15 modules, 22 differentially-expressed R gene transcripts, 58 PRR transcripts and 535 TF transcripts were involved (Supplementary Data S8). Moreover, nine modules were found to be significantly enriched in GO terms, particularly for biological processes closely related to the plant defense response (Supplementary Figure S10). One example was found in module 17, in which genes were mostly enriched in processes such as respiratory burst involved in defense response, systemic acquired resistance, salicylic acid biosynthetic process, regulation of plant-type hypersensitive response, innate immune response and negative regulation of programmed cell death (Supplementary Figure S10). We further investigated conserved domains of these species-specific modules. Consistent with the results from GO enrichment analysis, we found significant enrichments of domains related to signal transduction and immune response of alternative modules (Supplementary Data S9).

**Figure 7:**
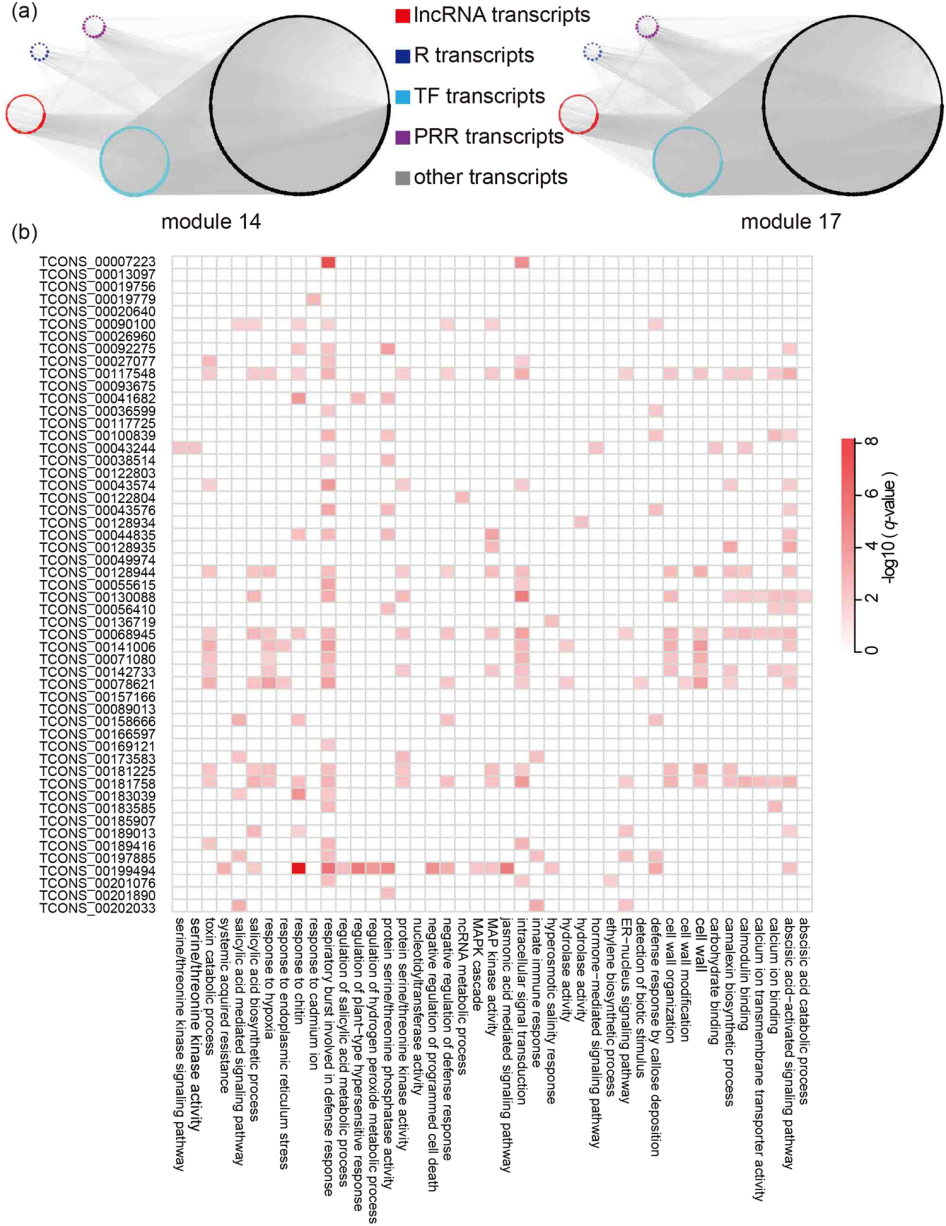
Network visualization of WGCNA modules and GO enrichments of genes associated with lncRNAs. (**a**) Network of module 14 and 17 on the basis of WGCNA analysis. (**b**) GO enrichment analysis of genes associated with lncRNAs within module 17.

Consistent with the distributions of R genes, PRR genes and TF genes, 79.4% (792 out of 998) of lncRNA transcripts were found within species-specific modules (Supplementary Figure S11a). Particularly, we found that some lncRNAs can be directly connected to R genes, PRR genes and TFs in co-expressed modules, suggesting a role of lncRNAs in regulating kiwifruit immune responses during Psa infection (Figs 7a and S11b). We inferred the function of a candidate lncRNA gene based on the protein-coding genes which were known to be in a specific functional process and were highly connected to the lncRNA in a co-expressed network, resulting in a set of lncRNAs in kiwifruit exhibiting potential functions associated with plant defense responses and signaling pathways (Fig. 7b and Supplementary Data S10). For example, lncRNA TCONS_0019494 from module 17 was involved in systemic acquired resistance, MAPK cascade and regulation of the plant-type hypersensitive response (Fig. 7b). Similarly, TCONS_00202033 in this module was found to be connected to innate immune response and the salicylic acid-mediated signaling pathway (Fig. 7b). In module 15, lncRNA TCONS_00076221 was identified as possibly contributing to plant PTI of the chitin catabolic process (Supplementary Data S10)^47^. Overall, the WGCNA results indicate a potential diverse role of lncRNAs in the responses of kiwifruit during Psa infection, including the pleiotropy of lncRNAs, while in contrast a set of lncRNAs can be commonly associated with a single biological process (Supplementary Data S10).

## Discussion

The advent of new techniques to examine the eukaryotic transcriptome has revealed the increasing complexity of eukaryotic gene expression^46^. Non-protein-coding genes including long non-protein-coding genes were reported to involve in regulation of gene expressions. In plants, lncRNAs have not been comprehensively identified and characterized^23, 26–30, 37^, even though it is known that their roles can be important in relation to multiple different biological processes^32–36^. In this study, we identified the lncRNAs which are particularly associated with the species-specific interaction of kiwifruit plants and their bacterial pathogen Psa which induces canker disease. Apart from the expression profile of lncRNAs, we characterized sets of co-expressed modules consisting of both protein-coding and lncRNA genes. It produce a systematic genetic background to explain the phenotypic variance of *Actinidia* taxa responsible for Psa infection^48, 49^.

Our results contribute to the increased understanding of the lncRNA repertoire in kiwifruit plants. Compared with protein-coding transcripts, lncRNAs identified in kiwifruit leaves during Psa infection were short, having lower GC content, fewer exons and shorter exons (Fig. 2d). The basic features and types of kiwifruit lncRNAs were consistent with those characterized in other plant species^28–31^. Recently, Tang *et al*.^37^ investigated lncRNA expression during fruit development and ripening in kiwifruit, and the same lncRNA types were found in our study. However, the proportion of each type presented in both studies is different (Supplementary Figure S12c), suggesting that alternative lncRNA types could have divergent functions associated with different biological processes (Fig. 2c)^37^. Only 13.8% (2049) of the lncRNAs identified in our study overlapped with those in the study by Tang *et al*.^37^, which implied the expression of lncRNAs may derive from different genomic locations or be due to alternative splicing. This indicated that lncRNAs expression is highly specific in particular biological processes and tissues.

Inferring lncRNA functions is critical to elucidate their roles in plant growth and adaptation, including the plant host–pathogen interaction. Transcriptome analysis of *Arabidopsis thaliana* infected by *Fusarium oxysporum* revealed that lncRNAs were related to disease defense, which was verified by T-DNA insertion and RNA-interference knockdown arrays^50^. In our case, we identified a group of lncRNAs which were relevant to kiwifruit defense responses to Psa infection (Supplementary Data S10). Functional analysis of lncRNA co-expressed protein-coding genes suggested these lncRNAs are related to multiple plant defense processes, including innate immune response, systemic acquired resistance, and salicylic acid-mediated defense (Supplementary Data S10 and Fig. 7b). Furthermore, lncRNAs can form extremely complex secondary structures with multi-loop-stem structures which may elaborate pleiotropy of lncRNAs. An increasing number of studies indicate that secondary structures of lncRNAs are more conserved and crucial for lncRNAs effects^34, 51^. This suggests that kiwifruit lncRNAs are potentially important players involved in host resistance/susceptibility to Psa infection, even though the real RNA motif recognition and dynamics of RNA confirmation requires subsequent experimental verification.

Psa infection of kiwifruit leaves causes dramatic alteration of the transcriptome in multiple biological processes, including carbohydrate metabolism, energy metabolism and photosynthesis (Supplementary Figure S4), to reassign nutrients in the host plants. However species-specific response patterns for both protein-coding genes and lncRNA genes were observed (Fig. 3), illustrating that varying strategies were utilized by different kiwifruit species to prevent the occurrence of bacterial canker disease. Within the plant–pathogen interaction pathway, we identified multiple species-specific expressed transcripts which regulated ROS and NO bursts, suggesting diversity in PTI of kiwifruit plants (Supplementary Data S4). Expression of R gene transcripts was also significantly changed in Ac (both AH and AJ), but no R transcripts were up-regulated in Ae and Aa during Psa infection (Supplementary Figure S12a). In contrast, significant up-regulation of PRR transcript expression was observed in Ac, Ae and Aa. These results suggest that Psa can suppress downstream processes of PAMP detection in Ac and cannot overcome the PTI of Ae and Aa (Supplementary Figure S12b). As aforementioned, the response of kiwifruit to Psa infection is a system-level process involving multiple biochemical pathways, which was revealed by WGCNA modules. These modules have both co-expressed protein coding genes and lncRNAs associated with PAMP detection, plant signaling processes, systemic acquired resistance, plant-type hypersensitive response and programmed cell death, which constituted the complex defense network (Supplementary Figure S11). These modules with sets of co-expressed genes also revealed species-specific expression patterns (Fig. 6), indicating diverse regulatory control mechanisms of different kiwifruit taxa in the face of Psa infection.

A central hypothesis addressed in this study is that diverse molecular patterns contribute to the various phenotypes of canker disease in different kiwifruit taxa. To clarify different expression, we investigate gene expression profiles during the infection by Psa of three representative kiwifruit taxa selected from the *Actinidia* genus. Currently, the kiwifruit cultivars are mostly derived from the species Ac in large-scale commercial planting, including cultivars with variable ploidy such as AH and AJ. The differences in gene expression and network effects within species between AH and AJ were not significantly different despite ploidy variation, but were highly specific at species-level, consistent with the evolutionary divergence of Ac, Ae and Aa^38^. Where the plant host and pathogen co-evolve within an agricultural ecosystem of monoculture, Psa can easily contact Ac and increase the evolution of specific effector genes which can overcome the PTI of Ac and further induce effector-triggered susceptibility (ETS). In contrast, Psa may not harbor specific effector genes to inhibit the PTI of either Ae or Aa (Supplementary Figure S12). Owing to variation in the phylogenetic background between species, PTI of both Ae and Aa can efficiently suppress colonization and reproduction of Psa during its initial infection of host plants, which is consistent with the weak fluorescence intensity and obscure symptoms of Ae and Aa observed at both 2 and 14 DPI (Fig. 1d).

Our results provide insights which will help to develop future strategies to prevent the kiwifruit bacterial canker disease caused by Psa. Given phylogenetic background of different species, the system-level differentiation of co-expressed gene modules is conserved and stably inherited Thus, interspecific hybridization is a promising approach to extend the genetic basis of the current cultivars, and breed new hybrid cultivars with high resistance to Psa. Recent studies revealed widespread natural hybridization between *Actinidia* taxa in the wild^52^, which could also provide opportunities to select natural hybrids as raw materials for resistance breeding. Alternatively, based on the species-specific gene modules related to plant immunity in our study, effective breeding programs can be further developed to create new resistant germplasm using advanced technologies such as the genome editing technique to directionally process hub-genes in the co-expressed networks (Supplementary Data S8 and 18). Functional annotations and verification of both protein-coding genes and lncRNAs have been initiating for further study.

## Materials and Methods

### Plant materials and incubation experiments

Psa strain C48 was chosen as the pathogenic agent for inoculation. This strain was isolated from a kiwifruit orchard in Anhui province, China, and belongs to the biovar 3 clade of the Psa phylogeny, members of which have high virulence and are responsible for the current worldwide outbreak of bacterial canker disease of kiwifruit^7^. To monitor Psa bacterial infection *in vivo* under long-wavelength ultraviolet (UV) light, a stable and broad-host-range plasmid vector (pDSK-GFPuv) strongly expressing GFPuv protein was transformed into the C48 strain using Bio-Rad MicroPulser (Bio-Rad, Hercules, CA, USA). The effect of the GFPuv-labeled strain on development of canker disease symptoms was further examined using incubation arrays by comparing the symptoms exhibited by a GFPuv-free strain to the same plant material of AH leaves. The fluorescence intensity in relation to the Psa bacterial population in kiwifruit leaf tissue was visualized using a Zeiss confocal laser scanning microscope 510Meta (Carl Zeiss Microscopy GmbH, Jena, Germany).

Seedlings of four kiwifruit materials (AH, AJ, Ae, and Aa) belonging to three different *Actinidia* species (Ac, Ae and Aa) were developed in a greenhouse in Spring 2015. Incubation experiments were performed using these plant materials in a plant growth chamber under controlled temperature (day: 25 °C, night: 20 °C) and humidity (70–100%). A bacterial suspension, containing approximately 10^8^ cells/mL Psa, was prepared from overnight culture and used for the inoculation. The bacterial suspension was injected into the petioles of three leaves from a single plantlet using sterilized syringes. To record the symptoms of each plant, the leaves were photographed under white light or long wavelength UV light (395 nm) at 0, 2 and 14 DPI. We then calculated the area of green fluorescence using ImageJ (http://imagej.net).

### RNA-Seq library preparation and sequencing

RNA was extracted from incubated leaves of each sample at 0 (before injection of bacteria), 2 and 14 DPI following the instructions provided with the HiPure Plant RNA Kits (Magen, Guangzhou, China). RNA quality was monitored on 1% agarose gels and RNA purity was checked using the NanoPhotometer® spectrophotometer (Implen, Munich, Germany). RNA concentration was measured using the Qubit® RNA Assay Kit in a Qubit® 2.0 Fluorimeter (Life Technologies, Carlsbad, CA, USA) and the integrity was assessed using the RNA Nano 6000 Assay Kit of the Bioanalyzer 2100 system (Agilent Technologies, Santa Clara, CA, USA). A total of 3 μg RNA per sample was used as input material for preparation of the RNA-seq libraries. Sequencing libraries were generated using a NEBNext® Ultra™ Directional RNA Library Prep Kit for Illumina® (New England Biolabs, Ipswich, MA, USA) following the manufacturer’s recommendations and index codes were added to attribute sequences to each sample. Library preparations were sequenced on an Illumina Hiseq 2000 platform (Illumina, San Diego, CA, USA) and 125 bp paired-end reads were generated. Each sample was sequenced with two biological replicates.

### Transcriptome assembly and annotations

Raw reads in fastq format were processed through in-house perl scripts by removing reads containing adapter or ploy-N and low quality reads. The STAR aligner (v2.4.1)^53^ was used to align paired-end reads to the combination of both the kiwifruit genome^2^ and Psa strain NZ13 as the genome references^7^. StringTie (v1.04)^51^ was used for assembly of the transcriptome for each sample, then cuffcompare^54^ was applied to compare the assembled transcriptome and the reference genome to optimize the annotations of protein-coding transcripts, and Annocript^40^ was used to annotate protein-coding transcripts. To verify the annotation of R and PRR genes, all transcripts were blasted to pfam database (http://pfam.xfam.org/) and filtered according to the conserved domains (NBS/LRR/TIR/RLK/CC) of R and PRR genes. To quantitate and compare expression of transcripts among samples, read numbers of each transcript were retrieved using RSEM (v1.2.17)^55^, and then used as input to calculate the expected number of fragments per kilobase of transcript sequence per million base pairs sequenced (FPKM).

### Identification and characterization of lncRNAs

To identify lncRNAs, we first retained transcripts more than 200 bp long or those with an open reading frame (ORF) less than 100 amino acids long, using in-house perl scripts. The transcripts annotated as protein-coding genes were further filtered. Based on the data of reference genome, we only retained transcripts with the class code of *j*, *i*, *o*, *u*, or *x* on the basis of cuffcompare results. The class code *j*, *i*, *o*, *u*, and *x* represent a potentially novel isoform, a transfrag falling entirely within a reference intron, generic exonic overlap with a reference transcript, an intergenic transcript and exonic overlap with reference on the opposite strand, respectively^56^. After further removing transcripts predicted to be housekeeping ncRNAs (tRNA or rRNA) based on the results of Annocript, we finally obtained transcripts which were predicted to be ncRNA by both CPC (with a cutoff CPC score <0)^57^ and CNCI (with a cutoff CNCI score <0)^54^. The classification of lncRNAs was performed based on originated regions of transcripts using in-house perl scripts. The criteria for classification of lncRNAs were as follows: (1) intergenic lncRNAs: located in intergenic regions and with a distance >500 bp to the closest protein-coding genes; (2) overlapping lncRNAs: located in protein-coding gene regions; (3) antisense lncRNAs: at least 50 bp overlap with their corresponding sense transcripts; (4) intronic lncRNAs: completely located within an intron of protein-coding genes. To compare features of protein-coding transcripts and lncRNA transcripts, we calculated transcript length, exon number and GC content using in-house perl scripts. To identify AS events in our transcriptomes, we used the BAM files from the STAR as the input files for the rMATs (v3.0.9)^58^. All parameters of rMATs were set as default values.

### Differential expression analysis and sample clustering

We used RSEM (v1.2.17) to calculate read counts from the output of BAM files^46^. Expression estimates in units of FPKM were obtained after normalizing the count matrices with sequencing depth and gene effective length. To calculate correlation between biological replicates, we used the *cor* function in R (v3.3.0, https://www.r-project.org/) to calculate correlation based on transcript FPKM. The combined FPKM matrix of both the protein-coding transcripts and the lncRNA transcripts was used as the input file for further principal component analyses using the *prcomp* function in R. To identify differentially-expressed transcripts, analysis of pairwise differential expression was performed with *DESeq2* ^59^ on the basis of the count matrices. We classified transcripts as differentially expressed if the adjusted *p-value* was less than 0.05 (FDR < 5%) and the moderate fold change >1. Sample clustering was performed using TM4 (v4.9)^60^ based on the expression matrix of protein-coding transcripts, lncRNA transcripts and all transcripts separately.

We selected genes from four representative gene families (*CNGCs*/*CPKs*/*MPKs*/*WRKYs*) in both kiwifruit and *Arabidopsis thaliana* (for CPKs family, sequences were originated from *Arabidopsis thaliana*, *Chlamydomonas reinhardtii*, *Coccomyxa subellipsoidea*, *Chlorella variabilis*, *Micromonas pusilla*, *Ostreococcus lucimarinus*, *Oryza sativa* ssp. *japonica*; *Ostreococcus tauri*, *Physcomitrella patens*, *Selaginella moellendorffii*, *Volvox carteri*) to investigate their phylogenetic relationships. The full-length amino acid sequences of all genes from both kiwifruit and other species were aligned and analyzed with ClustalW (the following alignment parameters: for pairwise alignment, gap opening 10.0 and gap extension 0.1; and for multiple alignment, gap opening 10.0 and gap extension 0.20)^61^ and the neighbor-joining tree (NJ) trees derived from 5000 replicates were constructed using MEGA 7 software^62^.

### Functional enrichment analysis

For GO enrichment analysis, the package *goseq* (v3.0)^63^ was used with default parameters. The results were filtered by FDR < 0.05. For KEGG enrichment analysis, KOBAS (v2.0, http://kobas.cbi.pku.edu.cn/) was used with default parameters filtered by FDR <0.05. For conserved domain enrichment analysis, *clusterProfiler* ^64^ in R was used with default parameters. The results were filtered by *p-value* < 0.05. Results of the KEGG pathway enrichment analysis were visualized using the iPATH 2 tool^65^ and VANTED 2^66^.

### Expression correlation analysis

Pairwise expression correlations between and within both the protein-coding and the lncRNA genes were calculated. Briefly, a table for different classes of gene pairs was created from the gene annotation table based on genomic position, including protein-coding–protein-coding, protein-coding–lncRNA and lncRNA–lncRNA gene pairs. For each pair of genes with non-null expression (FPKM >0), a non-parametric Spearman correlation was calculated on the basis of the FPKM matrix using R. Both *trans* (pairs consisting of genes located at a distance >2 Mb apart, or in different chromosomes) and *cis* (pairs consisting of genes located within a genomic window of 10 kb) correlations were computed. Correlations of lncRNA and mRNAs with randomly-shuffled gene pairs were used as control. The R package *ggplot2* (http://ggplot2.org/) was used to plot density histograms of the correlation coefficients rho (*r*_*s*_) for both *cis* and *trans* correlations^67^.

### Co-expression network analysis

We retrieved FPKM of differentially-expressed protein-coding transcripts and lncRNA transcripts that were expressed at an FPKM value of 0.1 or higher in at least one of the samples. This FPKM matrix included 24 columns and 9,698 rows, each column and row corresponding to one sample and one transcript respectively (Supplementary Data S6). Subsequent weighted gene co-expression network analysis was conducted using the R package WGCNA^45^. The retrieved FPKM matrix was used as the input file of WGCNA, and data filtering was performed to remove transcripts with too many missing values according to WGCNA cutoff threshold recommendations. Next we calculated a series soft thresholding power (from 1 to 20) following scale-free topology criteria and a soft thresholding power of 16 was chosen based on the approximate scale-free topology criteria. Then module detection was performed using a hierarchical clustering algorithm based on topological overlap values for all transcripts and finally modules of highly co-expressed transcripts were merged using a cutoff value of 0.25. The minimum size of each module was set as 30.

The eigengene of each module was calculated and the Pearson correlation coefficients between module eigengenes and traits (infected leaf area, sampling points and three species phenotypes) were counted using TM4 (v4.9)^60^. To screen trait-associated modules, we set a cutoff *p-value* of 0.05 (Fig. 6). To investigate the correlation of different modules, hierarchical clustering for all module eigengenes was conducted using TM4 (v4.9)^60^. We used in-house perl scripts to find R, PRR, TF and lncRNA transcripts within modules, and performed GO and conserved-domain enrichment analysis for protein-coding transcripts within each module to study the functional characteristics of each module. To directly examine the relationships between transcripts within the same module, we visualized the network of each module using Cytoscape 3.3.0^68^. The potential function of each lncRNA was inferred on the basis of protein-coding transcripts linked directly to the module network^48^.

### Experimental validation of protein-coding genes and lncRNA genes

All primers were designed using the Primer3Plus software online (http://www.bioinformatics.nl/cgi-bin/primer3plus/primer3plus.cgi) and synthesized commercially (Sangon Biotech Co., Ltd., Shanghai, China) (Supplementary Table S5). The cDNA of samples was prepared with the One-step gDNA removal and cDNA synthesis supermix kit (TransGen Biotech Co., Ltd., Beijing, China), and was then used as the input for all PCR experiments. QPCR was carried out in a total volume of 20 µL, containing 10 µL of Tip Green qPCR SuperMix (TransGen Biotech Co., Ltd.), 0.2 µM of each primer, 1 µL of 1:5 diluted cDNA and 8.2 µL ddH_2_O. Thermal cycling consisted of a hold at 94 °C for 30 s, followed by 40 cycles of 94 °C for 5 s, and 60 °C for 30 s. The temperature was then gradually raised, by 0.5 °C every 10 s, to perform melting-curve analysis. Each sample was amplified in triplicate, and all PCR reactions were performed on the LightCycler 480 (Roche, Basel, Switzerland). The ΔΔCt method was employed with *Achn107181* (kiwifruit Actin gene) and *Achn381211* (protein phosphatase 2 A, PP2A-like gene) as endogenous control^69^.

### Availability of supporting data

The raw reads of all samples are publicly available in the National Center for Biotechnology Information (NCBI) database under bioproject ID PRJNA328414, sample accession IDs SRS1552843, SRS1552846-SRS1552860, SRS1552862-SRS1552865, and SRS1552867-SRS1552876. All the source codes used for the analysis are provided at https://github.com/wangzupeng13/Perl-scripts-of-lncRNA-transcriptome.

## Electronic supplementary material

Supplementary materials and methods, supplementary Tables (S1-S6) and supplementary Figure S1-S11 and with legends

Supplementary Data S1

Supplementary Data S2

Supplementary Data S3

Supplementary Data S4

Supplementary Data S5

Supplementary Data S6

Supplementary Data S7

Supplementary Data S8

Supplementary Data S9

Supplementary Data S10
