# Supplementary material for: Whole transcriptome sequencing of Pseudomonas syringae pv. actinidiae-infected kiwifruit plants reveals species-specific interaction between long non-coding RNA and coding genes
Source: Sci Rep. 2017 Jul 7;7:4910. doi: 10.1038/s41598-017-05377-y (PMC5501815; doi:10.1038/s41598-017-05377-y)
Supplement: Supplementary file 1 — Supplementary materials and methods, supplementary Tables (S1-S6) and supplementary Figure S1-S11 and with legends [file 41598_2017_5377_MOESM1_ESM.pdf]

Whole transcriptome sequencing of *Pseudomonas syringae* pv. *actinidiae*-infected kiwifruit plants reveals species-specific interaction between long non-coding RNA and coding genes

Authors: Zupeng Wang, Yifei Liu, Li Li, Dawei Li, Qiong Zhang, Yangtao Guo, Shuaibin Wang, Caihong Zhong, Hongwen Huang

Supplementary Information

Supplementary Materials and Methods

Plant materials. In the present study, we employed three representative *Actinidia* taxa (Ac: *A. chinensis*; Ae: *A. eriantha*; Aa: *A. arguta*) to investigate their resistance/susceptibility in the face of Psa infection. Two additional cultivars with different ploidy, 'Hongyang' (AH: diploid) and 'Jinyan' (AJ: tetraploid) within the Ac taxon were also included. These three species belong to three distinct clades within the phylogenetic tree of the *Actinidia* genus, and comparatively Aa is more phylogenetically distinct from Ac than is Ae [1]. The vast majority of commercial kiwifruit plantings were developed from the species Ac, representing a very small fraction of the entire genetic diversity exhibited by wild kiwifruit plants [2]. Domestication and commercialization of both Ae and Aa have just begun although some of their fruit traits are highly attractive for breeding, such as high resistance to adverse conditions, high vitamin C content, easy to peel fruit skin and good storage [2].

Library preparation for lncRNA sequencing. A total of 3 μg RNA per sample was used as

input material for the construction of an RNA-Seq library. Sequencing libraries were generated using a NEBNext® Ultra™ Directional RNA Library Prep Kit for Illumina® (New England Biolabs, Ipswich, MA, USA) following the manufacturer's recommendations, and index codes were added to attribute sequences to each sample. Briefly, fragmentation was carried out using divalent cations under elevated temperature in NEBNext First Strand Synthesis Reaction Buffer (5×). First strand cDNA was synthesized using random hexamer primers and M-MuLV reverse transcriptase (RNaseH-). Second strand cDNA synthesis was subsequently performed using DNA Polymerase I and RNase H. In the reaction buffer, dTTP was replaced by dUTP in the dNTP mixture. Remaining overhangs were converted into blunt ends by the action of exonuclease/polymerase. After adenylation of 3' ends of DNA fragments, a NEBNext adaptor with hairpin loop structure was ligated to prepare for hybridization. In order to select cDNA fragments ideally of 150–200 bp in length, the library fragments were purified using the AMPure XP system (Beckman Coulter, Brea, CA, USA). Then 3 μL USER Enzyme (New England Biolabs) was used with size-selected, adaptor-ligated cDNA at 37°C for 15 min followed by 5 min at 95°C before PCR. PCR was performed with Phusion High-Fidelity DNA polymerase, Universal PCR primers and Index (X) Primer. Finally, products were purified (AMPure XP system) and library quality was assessed on the Agilent Bioanalyzer 2100 system.

Clustering and sequencing. Clustering of the index-coded samples was performed on a cBot Cluster Generation System using a TruSeq PE Cluster Kit v3-cBot-HS (Illumina, San Diego, CA, USA) according to the manufacturer's instructions. After cluster generation, the library

preparations were sequenced on an Illumina Hiseq 2000 platform and 125 bp paired-end reads were generated.

Transcriptome assembly and annotation. After quality control of raw data, clean RNA-seq reads were aligned to the combined genome (including the kiwifruit genome and the Psa genome of NZ13) with STAR (v2.4.1c) [3]. The default parameters of STAR were optimized: --outSAMstrandField intronMotif; --outFilterIntroMotifs; --twopassMode. After alignment we employed StringTie (v1.04) to assemble the transcriptome for each sample separately based on the SAM outputs of STAR [4]. To obtain more accurate results, we set the parameter *–j* of StringTie to the value 2. To optimize structures of protein-coding genes and retain positional information from the reference genome, we compared the GTF file of each transcriptome and the GTF file of the reference genome using cuffcompare with default parameters [5]. We then used the Annocript pipeline to annotate protein-coding transcripts of our transcriptomes [6]. The Annocript pipeline is a comprehensive and integrated collection including several programs [6], as follows, together with running parameters: blastx (ncbi-blast-2.2.30+, parameters: -evalue 1e-5, -word_size 4, -num_descriptions 5, -num_alignments 5, blasting to SwissProt and UniRef90 (201504)); blastn (ncbi-blast-2.2.30+, parameters: -evalue 1e-5, -num_descriptions 1, -num_alignments 1, blasting to Rfam) and rpblast (ncbi-blast-2.2.30+, parameters: -evalue 1e-5 -num_descriptions 20 -num_alignments 20, blasting to Conserved Domain Database of NCBI) [7].

Inferring functions of lncRNAs. We first exported network results of all WGCNA modules [8]. Next we retained lncRNAs within each module and retrieved transcript ids of

protein-coding transcripts which were directly connected to the same lncRNA which we saved as a separate file. At the same time we retained the sub-network of each lncRNA from the whole network of the corresponding module and performed GO enrichment analysis and conserved domain enrichment analysis for protein-coding gene sets associated with lncRNAs using clusterProfiler in R (v3.3.0) with default parameters [9]. To visualize the sub-network of each lncRNA, we used Cytoscape 3.3.0 to construct the network [10].

References

1. Li, J., Huang, H. & Sang, T. Molecular phylogeny and infrageneric classification of *Actinidia* (Actinidiaceae). *Syst. Bot.* **27,** 408–415 (2002).

2. Huang, H. *The genus Actinidia, a world monograph*. *Science Press*, BeiJing (2014).

3. Dobin, A. *et al.* STAR: Ultrafast universal RNA-seq aligner. *Bioinformatics* **29,** 15–21 (2013).

4. Mihaela Pertea, J. T. M. S. L. S. StringTie enables improved reconstruction of a transcriptome from RNA-seq reads. *Nat. Biotechnol.* **33,** 290–295 (2015).

5. Trapnell, C. *et al.* Transcript assembly and quantification by RNA-Seq reveals unannotated transcripts and isoform switching during cell differentiation. *Nat. Biotechnol.* **28,** 511–515 (2010).

6. Musacchia, F., Basu, S., Petrosino, G., Salvemini, M. & Sanges, R. Annocript: A flexible pipeline for the annotation of transcriptomes able to identify putative long noncoding RNAs. *Bioinformatics* **31,** 2199–2201 (2015).

7. Altschul, S. F. *et al.* Gapped BLAST and PSI-BLAST:a new generation of protein database search programs. *Nucleic Acids Res* **25,** 3389–3402 (1997).

8. Langfelder, P. & Horvath, S. WGCNA: an R package for weighted correlation network analysis. *BMC Bioinformatics* **9,** 559 (2008).

9. Yu, G., Wang, L. G., Han, Y. & He, Q.Y. clusterProfiler: an R package for comparing

biological themes among gene clusters. *Omi. a J. Integr. Biol.* **16,** 284–287 (2012).

10. Demchak, B. *et al.* Cytoscape: the network visualization tool for GenomeSpace workflows. *F1000Research* **3,** 151 (2014).

Supplementary Table S1. The *Actinidia* materials and species used in our analysis.

Species name	Material name	Sampling point	Replicate 1	Replicate 2
Actinidia chinensis (Ac)	AH	0 DPI	AH-1-1	AH-2-1
	AH	2 DPI	AH-1-2	AH-2-2
	AH	14 DPI	AH-1-3	AH-2-3
	AJ	0 DPI	AJ-1-1	AJ-2-1
	AJ	2 DPI	AJ-1-2	AJ-2-2
	AJ	14 DPI	AJ-1-3	AJ-2-3
Actinidia eriantha (Ae)	Ae	0 DPI	Ae-1-1	Ae-2-1
	Ae	2 DPI	Ae-1-2	Ae-2-2
	Ae	14 DPI	Ae-1-3	Ae-2-3
Actinidia arguta (Aa)	Aa	0 DPI	Aa-1-1	Aa-2-1
	Aa	2 DPI	Aa-1-2	Aa-2-2
	Aa	14 DPI	Aa-1-3	Aa-2-3

The last two columns of first biological replicate and the second biological replicate respectively.
DPI: days post incubation.

Supplementary Table S2. Summary of sequencing for 24 RNA-seq libraries.

Sample name	Total reads	GC content	Q20	Mapping to kiwifruit	Mapping to Psa
AH-1-1	95,015,514	0.4248	0.9621	86.16%	0.00%
AH-1-2	101,575,728	0.4212	0.9653	85.78%	0.47%
AH-1-3	95,662,314	0.431	0.9646	86.20%	0.28%
AH-2-1	88,694,186	0.4262	0.9624	86.93%	0.00%
AH-2-2	100,215,130	0.4261	0.967	85.39%	0.25%
AH-2-3	100,472,526	0.4334	0.9661	88.62%	0.15%
AJ-1-1	99,104,350	0.4417	0.9649	87.50%	0.00%
AJ-1-2	94,450,724	0.4273	0.9658	86.08%	0.08%
AJ-1-3	102,970,044	0.4262	0.962	78.31%	0.32%
AJ-2-1	102,048,602	0.4363	0.9626	85.30%	0.00%
AJ-2-2	94,064,890	0.4316	0.9614	85.92%	0.14%
AJ-2-3	98,841,800	0.4378	0.9624	85.70%	0.09%
Ae-1-1	93,432,478	0.4255	0.9615	84.41%	0.00%
Ae-1-2	90,264,824	0.4239	0.9634	83.94%	0.08%
Ae-1-3	97,814,174	0.426	0.9629	84.62%	0.73%
Ae-2-1	94,936,626	0.4223	0.9662	84.08%	0.00%
Ae-2-2	89,162,274	0.4246	0.9621	84.00%	0.31%
Ae-2-3	95,199,630	0.4226	0.9597	83.69%	1.40%
Aa-1-1	96,509,688	0.4221	0.9667	75.97%	0.00%
Aa-1-2	93,176,626	0.4255	0.9669	76.65%	0.10%
Aa-1-3	93,992,916	0.4211	0.9678	77.00%	0.21%
Aa-2-1	97,259,642	0.4321	0.9619	75.98%	0.00%
Aa-2-2	99,141,226	0.4269	0.9681	76.76%	0.11%
Aa-2-3	90,110,246	0.4318	0.9696	76.49%	0.15%

"Total reads" were the numbers of raw data of samples; "GC content" represents value of each sample based on the clean data; "Mapping to kiwifruit" represents the proportion of reads mapping to the kiwifruit genome; "Mapping to Psa" represents the proportion of reads mapping to the Psa strain NZ13 genome.

Supplementary Table S3. Average numbers of differential expressed protein-coding and lncRNA transcripts at different sampling points.

Transcript type	Sampling point	Average number of differential expressed transcripts	percent
protein-coding transcript	0 DPI	17599	14.8%
	2 DPI	13862	11.6%
	14 DPI	7485	6.3%
lncRNA transcript	0 DPI	172	1.2%
	2 DPI	194	1.3%
	14 DPI	134	0.9%

From the first column to the fourth column represent transcript type, sampling point and average number of differential expressed transcripts, the proportion of average number of differential expressed transcript.

Supplementary Table S4. The numbers of KO entry mapped to the metabolic pathways, the regulatory pathways and the biosynthesis of secondary metabolites respectively on the basis of differential expressed transcripts.

Species	Sample	Stage	Number of KO entry mapped to Metabolic pathways	Number of KO entry mapped to Regulatory pathways	Number of KO entry mapped to Biosynthesis of secondary metabolites
			up-regulated<br>down-regulated	up-regulated<br>down-regulated	up-regulated<br>down-regulated
Ac	AH	0-2 DPI	111	31	44
			84	20	34
		2-14 DPI	128	30	64
			68	31	35
	AJ	0-2 DPI	143	50	66
			169	66	58
		2-14 DPI	187	52	83
			44	24	16
Ae	Ae	0-2 DPI	130	22	63
			112	20	33
		2-14 DPI	13	7	7
			7	4	4
Aa	AJ	0-2 DPI	44	9	10
			9	5	4
		2-14 DPI	35	12	8
			26	8	14

up-regulated and down-regulated mean the expression of transcripts were increased and decreased respectively.
KO: the serial number of terms in the KEGG database.

Table S5. Primers used for RT-PCR and qRT-PCR analysis

Gene id	Primers(5'--3')	Correlation between qPCR and RNA-seq
TCONS_00203851	GTCACCTTCTACCGTCTAT<br>ATGCTAATGCTCCTCTGC	0.80
TCONS_00131511	CAATGGCGTTTATGAGGG<br>CGTTGGATGGAGGTGAAG	0.95
TCONS_00045970	TGAATCGGTGACGGAGGT<br>ACAGCGGCAACGAAGAAG	0.84
TCONS_00024491	ATCCCTCTTTTCCCTAC<br>CCATTCAATAAGCCAAC	0.90
TCONS_00022101	ATGGTGGTAATCTGGTGA<br>CAACTGAAATAGGGACAA	0.91
TCONS_00017053	CCATCCCAGCATTCTTCA<br>TCGTTTACAACCCGCACT	0.84
Achn322281	GGTTCGGGTGGTTTCATA<br>TTCCCTGTGGCAGTCTTT	0.74
Achn314751	TACCCACAAAGAACAACAGA<br>CAACATTAGGCACCAAGC	0.90
Achn296121	CAGTGGCTGGAAGTTGAA<br>ATGGGAGGTGGCTAAGAT	0.75
Achn267731	GTTCAAAGAGGGTGAGCCA<br>GGAAGGTCCAAGACATAGAGG	0.90
Achn245451	GGGCTGGCACAGACTACTA<br>TCGCTCCTGAGAACTTGATA	0.97
Achn154321	GCCGTTCCAGCCATACAG<br>ACAGCCGCTTCTCCTCGTC	0.94
Achn153111	GTGGGTGAGATTGTTGGC<br>AGCAGATTCAGCAGGGTT	0.75
Achn144351	GAGCCCATTTACCCACCG<br>CTCGCAGAAGCAGTCCATCA	0.90
Achn079081	CCTCTGCCTTACTCACCACA<br>AAGCGAGCACATCCACGA	0.92
Achn077461	GTCTACACGGCTTTGCTG<br>AACCCTCTATCTCATCTTTCC	0.94
Achn047581	GCCCTTTCGCTTCGCTTAT<br>TTGCCACTGCGGTCCCTA	0.87
Achn036661	ACAGGCTCGGTTTGGATTT<br>CTGGTTCTTCGCAAGGTTC	0.87
Achn019431	GGAAGGACTATGTGGACCCG<br>CCAGGAACAGCCCGAAC	0.85
Achn026931	TTGTTGGCAGATTTGAGA<br>CAGCAGGGTTTGTAGCA	0.85
Achn028481	AAATCCAAAGTTACCACCCT<br>CTCCCACTCCTTCACAGC	0.88
Achn039801	CGGAAACACTCAACTACCA<br>CCAATCACGCACGCTA	0.80
Achn053521	CAAATCTGTGGGCTCTAT<br>TCCTCACTTGCCTTCTG	0.84
Achn132971	TCTGAGGGTGGTTTCG<br>TCTTGTCTCCTGCTTGG	0.88
Achn155671	AGGCGAGTTTGAGTTGGT<br>CTTGTCGTCGGGCATT	0.89
Achn176331	GCGTGGGTGTCTACTTC<br>GCCTGGTTCTGTTTCG	0.81

Achn188591	AATGGGTGTTTCCAGGTC<br>GGACAAAGGTGCCAATAA	0.90
Achn192771	GGACATTGGTCGGTTTA<br>ATTCTGCTCGGGCTTT	0.83
Achn208471	CACAGGCATCCACAGC<br>GACGACGAAGCGAAGTA	0.95
Achn252871	TTAACAGGTCCTCATATCCC<br>CTCAGGTTTCTTCCTCCAA	0.88
Achn280231	TTCAACTCGGTCAAGGATAT<br>CGAAGCAAATGAGCCAC	0.90
Achn287671	GGCGAGGGTGCTACAA<br>GGTTCGTCTTCGGTCAT	0.81
Achn328501	CACCTCCTTATTACCATCA<br>GAGCAGAGCCTCAAACT	0.92
Achn330331	AGAGGCTGTGAACAAGGTG<br>GGTTGGAGTGGGCGTA	0.97
Achn331061	CATCAAAGCCGCAGTTA<br>GAGCCTCATCAAGGAAAG	0.94
Achn339761	GGCTACTCAATGAACGCTAT<br>CCACCGTCCCTTACAAA	0.80
Achn347161	CAGGGCTGTTCTTCCA<br>GCATTAGGCACCATTTC	0.92
Achn389521	ACCCACCGCTACTGAC<br>AGACCCGCTAACGAAG	0.91
TCONS_00045978	TTTACGAGGCTGTGATG<br>CGCAACGGAGGTTAGAT	0.92
TCONS_00182693	TTGTGCTTGCGTTGAT<br>AATGTGCCAAAGGAGT	0.91
TCONS_00195214	GACTCCTTTGGCACAT<br>GGACCGACCTCTATCA	0.84
XLOC_008312	GCCTAAAGAGCCACCC<br>GCTTCAGCCTCGTCCT	0.86
XLOC_038103	GGGCAATAAGGCTGTCG<br>TCACGGGCACTGATAACG	0.97
XLOC_041968	GGATGAACCACCCTACC<br>AGCACATGAAGCCACAG	0.94
XLOC_059159	TCTTGATTGGGAAACC<br>AACGACGGGATGAGTA	0.86
XLOC_061955	AAAGGATAAGGGCAAGG<br>AACCGATGACCAAAGT	0.81
XLOC_068977	GATAGTTCTCGCCTTTG<br>TACCCAGTCATTGTTCAT	0.90
XLOC_072856	AGAAAGGTTATGCCAATG<br>TCACGACCAGGAAAGC	0.84
XLOC_074907	GTGGGCTTCTGAGTGT<br>GGTGAATAAGGGTTGAG	0.93
XLOC_081091	CGCTCCCTATGTTCTG<br>GTCACCTGCCCTCAAT	0.96
Achn107181	TGAGAGATTCCGTTGCCCAGAAGT<br>TTCCTTACTCATGCGGTCTGCGAT	-
PP2A	GCAGCACATAATTCCACAGG<br>TTTCTGAGCCCATAACAGGAG	-

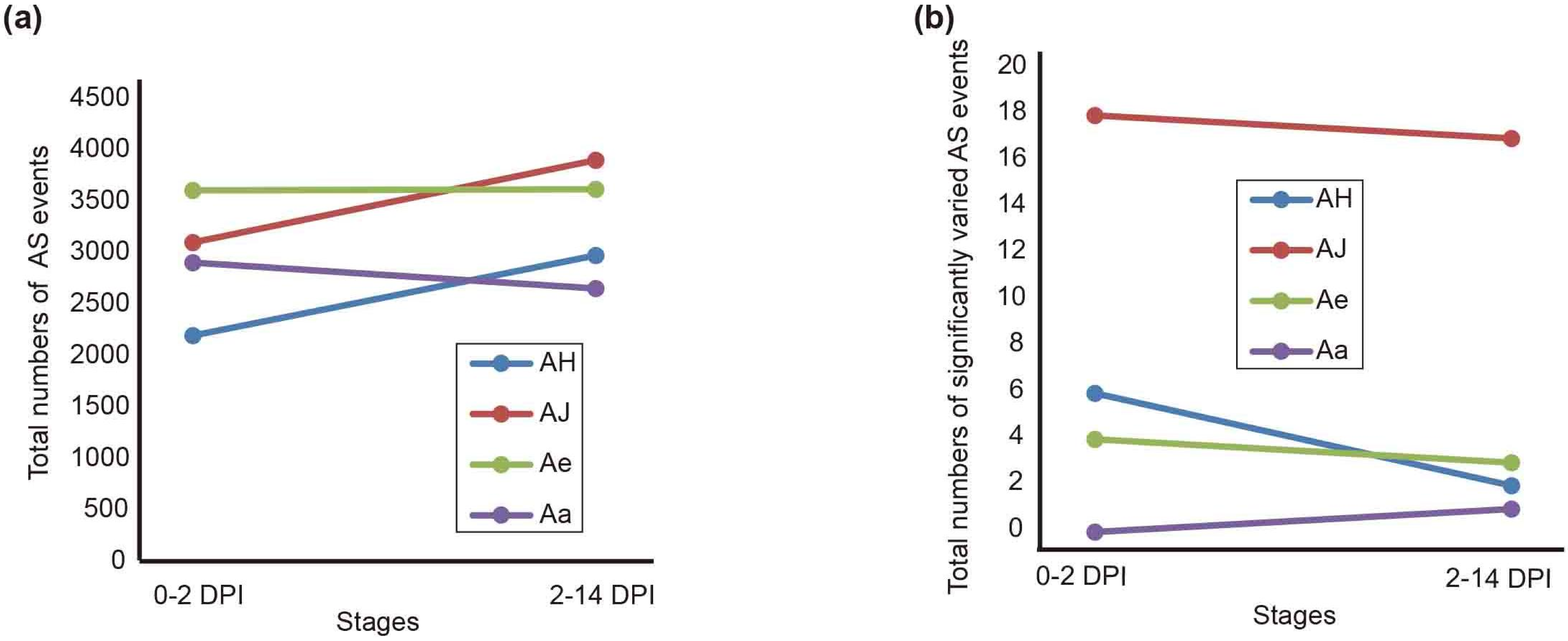

Supplementary Figure S1 Changes in the total numbers of AS events and significantly varied AS events during Psa invasion. (a) Change of the total number of AS events. (b) Change of the total number of significantly varied AS events.

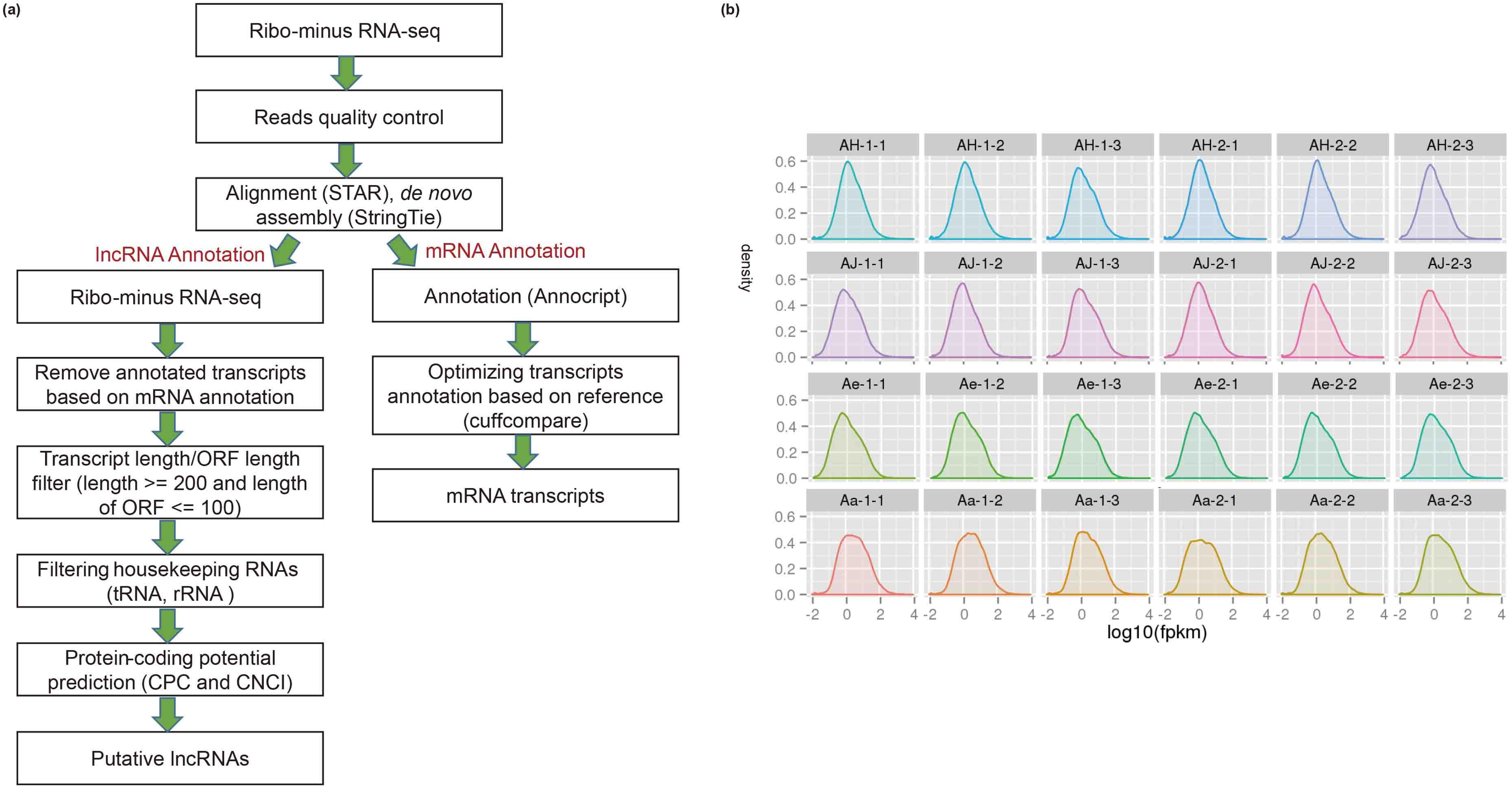

Supplementary Figure S2 Pipeline for transcriptomic data analysis and gene annotations. (a) Details of RNA-seq data analysis for both protein-coding and lncRNA transcripts. (b) Expression of all lncRNAs identified for RNA-seq library construction.

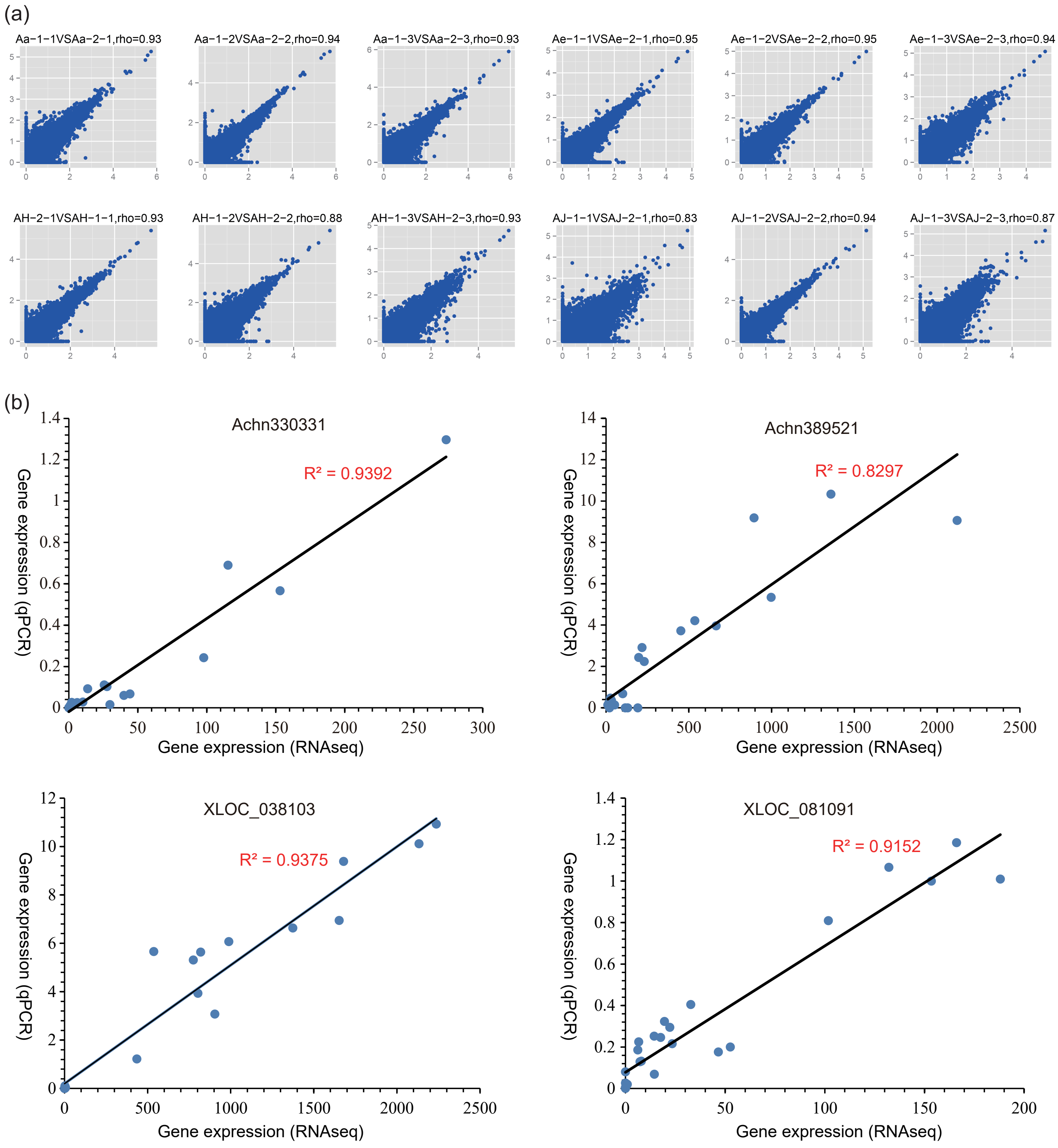

Supplementary Figure S3. Evaluation and qPCR verification of the RNA-seq data. (a) Scatter diagram indicating significant correlations between biological replicates for each sample. (b) Examples of Comparison of gene expression values between the qPCR experimental results and the RNA-seq data. The kiwifruit actin gene and protein phosphatase 2A gene were used as the reference gene for qPCR experiments.

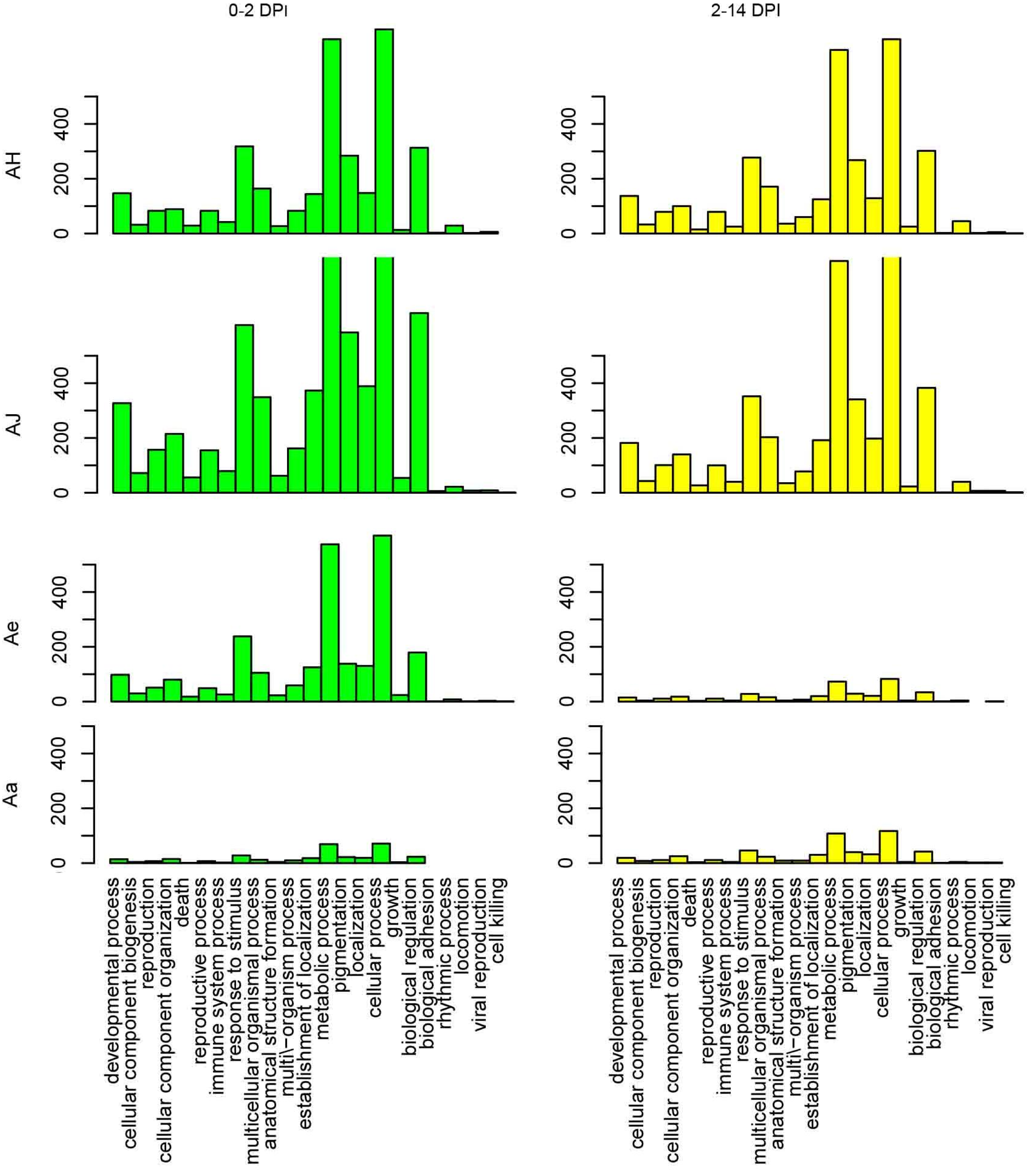

Supplementary Figure S4 GO enrichment analysis of differentially-expressed transcripts for the four *Actinidia* samples examined during Psa infection. The first stage (0–2 DPI) and the second stage (2–14 DPI) represents comparisons between the 0 and 2 DPI and between 2 and 14 DPI sampling time-points respectively. The height of each bar represents the number of transcripts associated with the corresponding GO term.

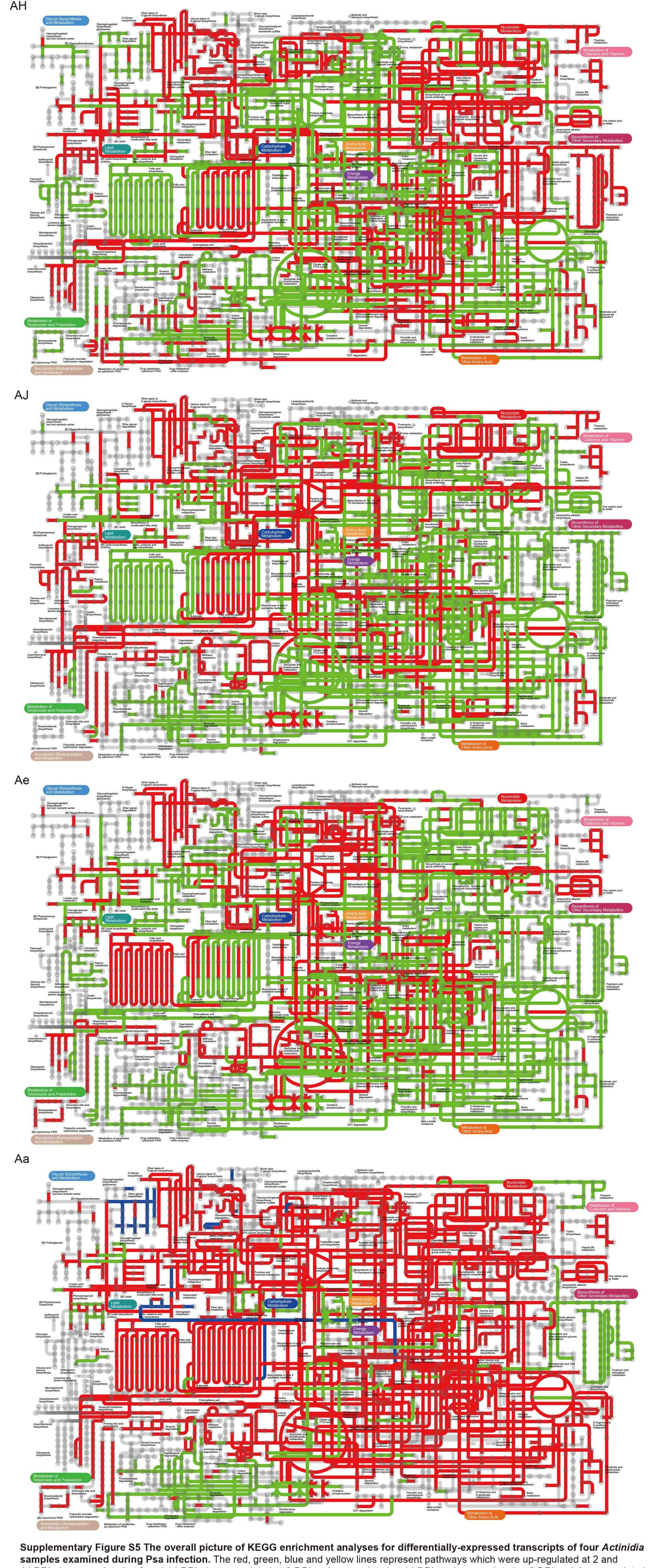

Supplementary Figure S5 The overall picture of KEGG enrichment analyses for differentially-expressed transcripts of four *Actinidia* samples examined during Psa infection. The red, green, blue and yellow lines represent pathways which were up-regulated at 2 and 14 DPI, down-regulated at 2 and 14 DPI, down-regulated at 2 DPI and up-regulated at 14 DPI, and up-regulated at 2 DPI and down-regulated at 14 DPI, respectively.

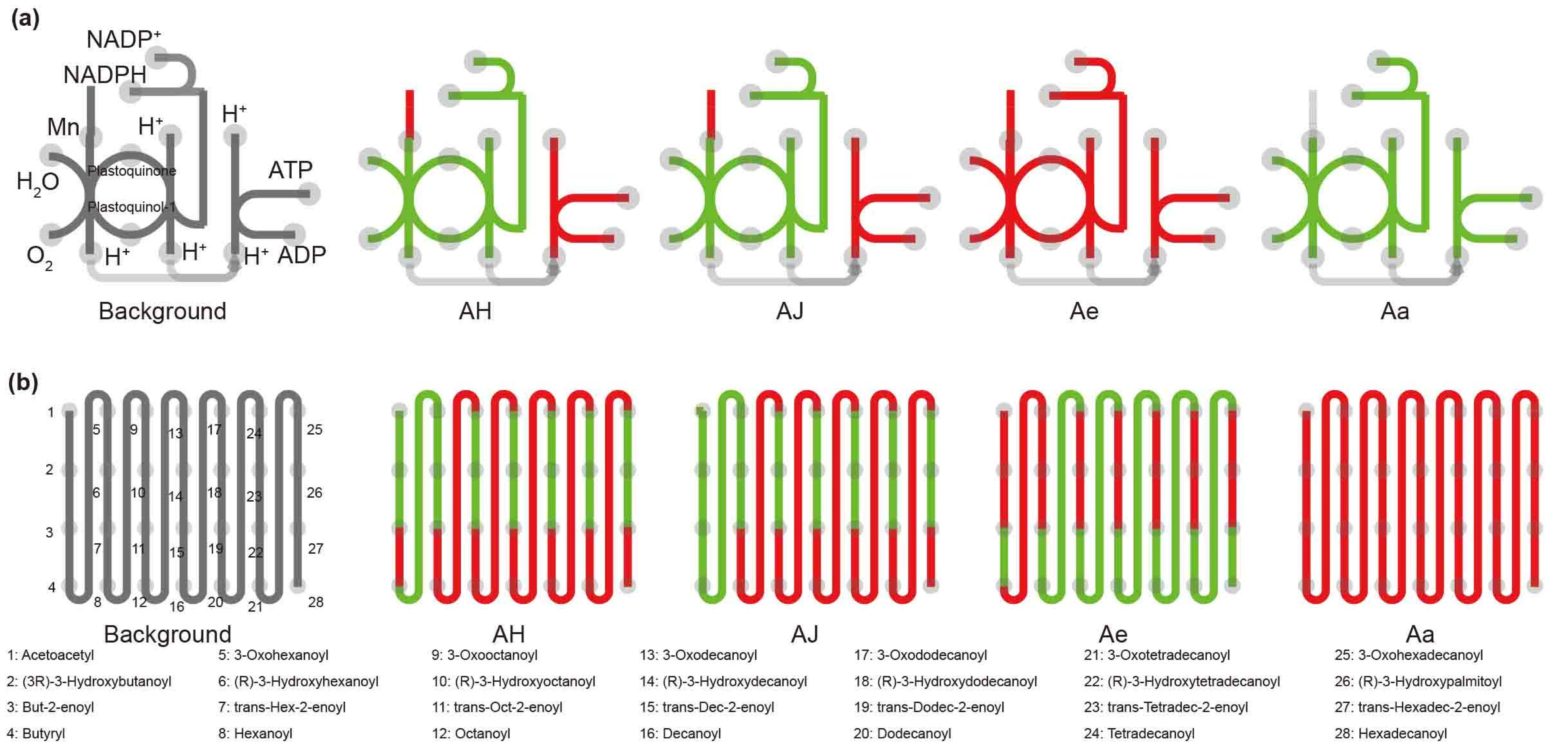

Supplementary Figure S6 Differentially-expressed transcripts involved in the photosynthesis. (a) **photosynthesis** (b) the fatty acid metabolism. The red, green, blue and yellow lines represent pathways which were up-regulated at 2 and 14 DPI, down-regulated at 2 and 14 DPI, down-regulated at 2 DPI and up-regulated at 14 DPI, and up-regulated at 2 DPI and down-regulated at 14 DPI, respectively.

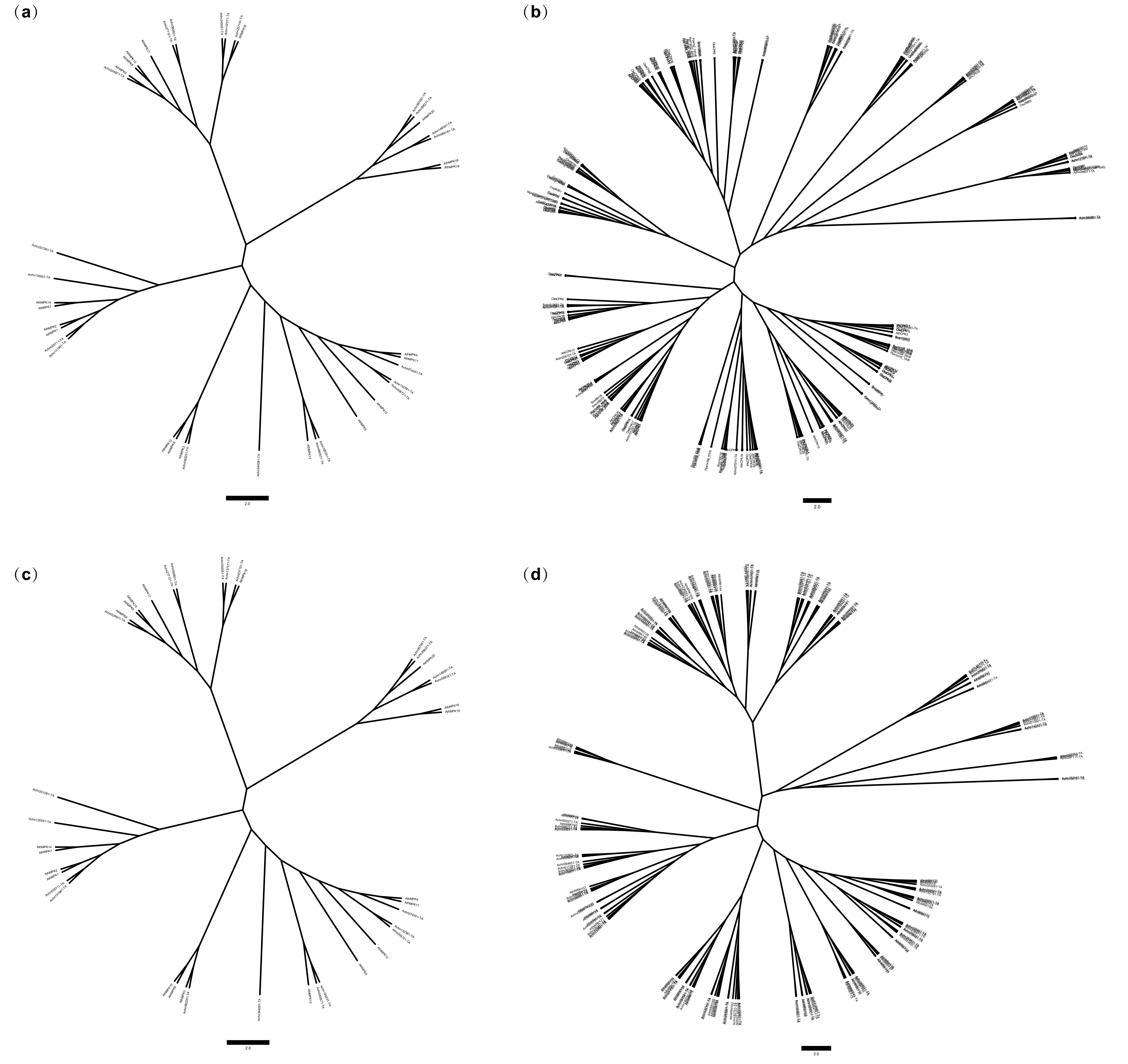

Supplementary Figure S7 Relation trees of four gene families (CNGCs/CPKs/MPKs/WRKYs) in kiwifruit and *Arabidopsis thaliana*. The full-length amino acid sequences of all genes from different species were aligned and analyzed with ClustalX. A species acronym identifies the origin of each protein (Ath, Arabidopsis thaliana; Cre, Chlamydomonas reinhardtii; Csu, Coccomyxa subellipsoidea; Cva, Chlorella variabilis; Mpu, Micromonas pusilla; Olu,Ostreococcus lucimarinus; Osa, Oryza sativa ssp. japonica; Ota, Ostreococcus tauri; Ppa, Physcomitrella patens; Smo, Selaginella moellendorffii; Vca, Volvox carteri; Achn, Actinidia). (a) CNGCs. (b) CPKs. (c) MPKs. (d) WRKYs.

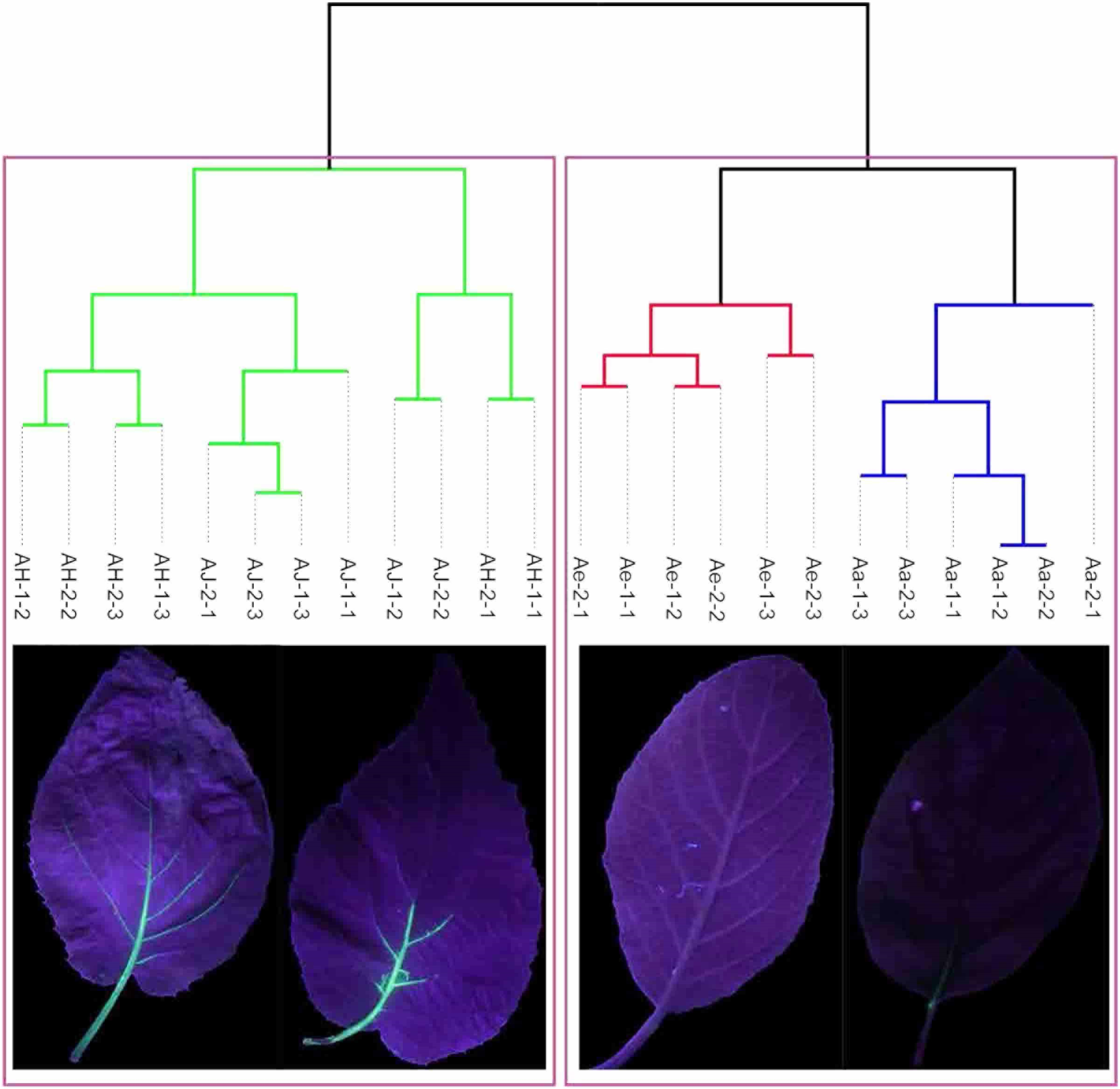

Supplementary Figure S8 Clustering of samples on the basis of transcripts involved in the plant–pathogen interaction pathway. The corresponding leaf symptoms of four samples at 14 DPI are shown below the dendrogram.

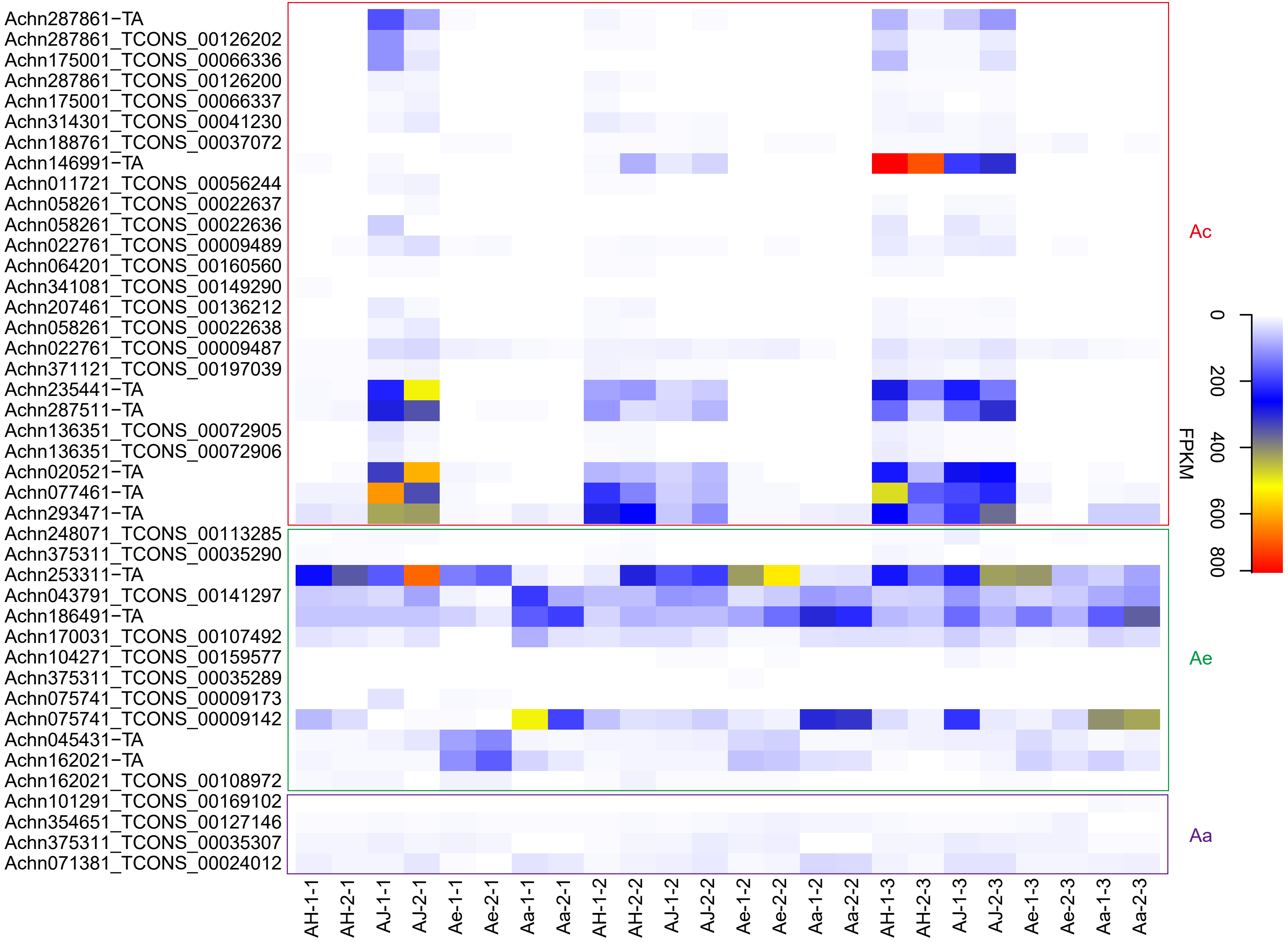

Supplementary Figure S9 Heatmap of species-specific expressed transcripts involved in the plant–pathogen interaction pathway.

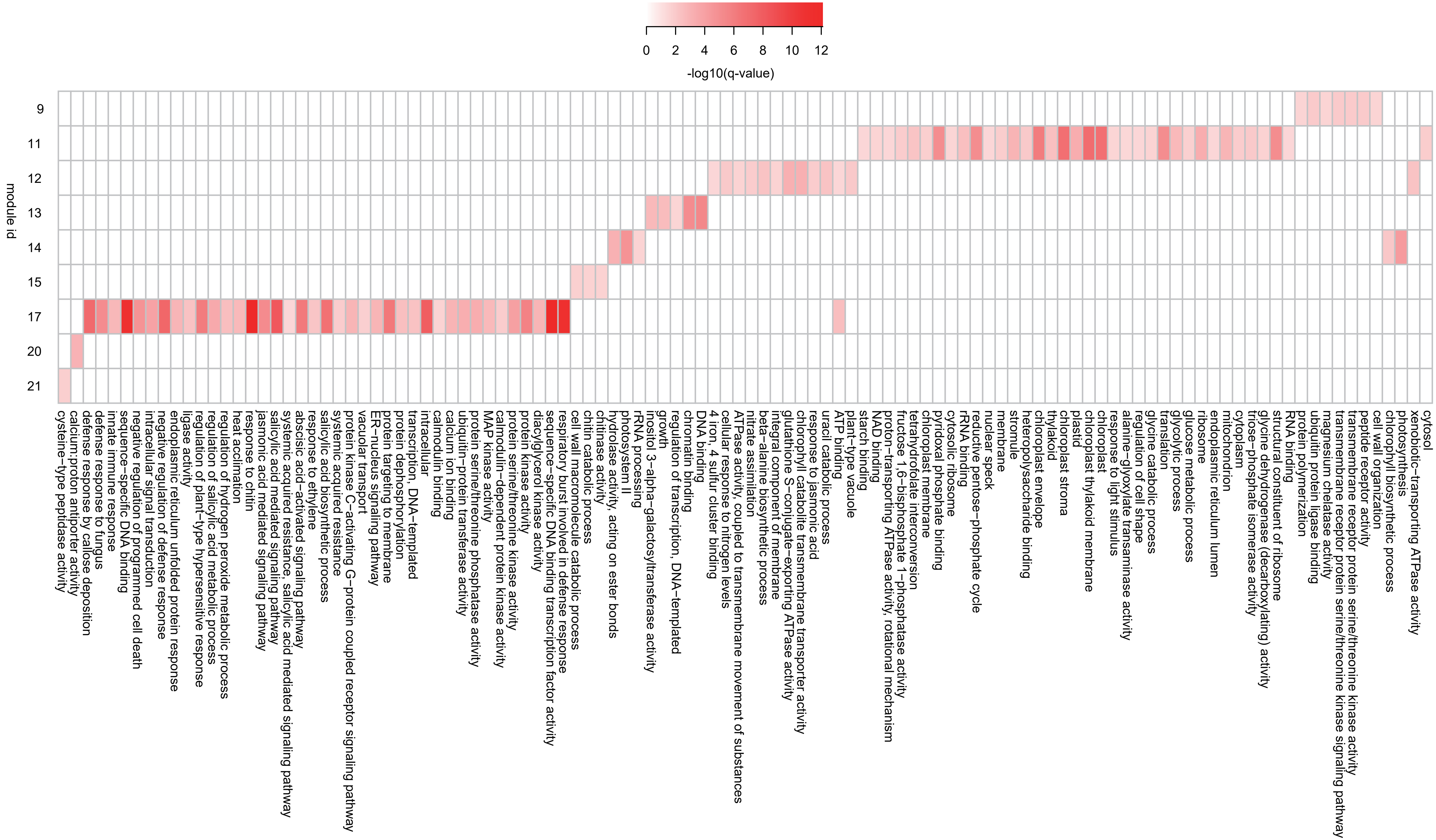

Supplementary Figure S10 GO enrichment analysis of protein-coding genes within each species-specific module.

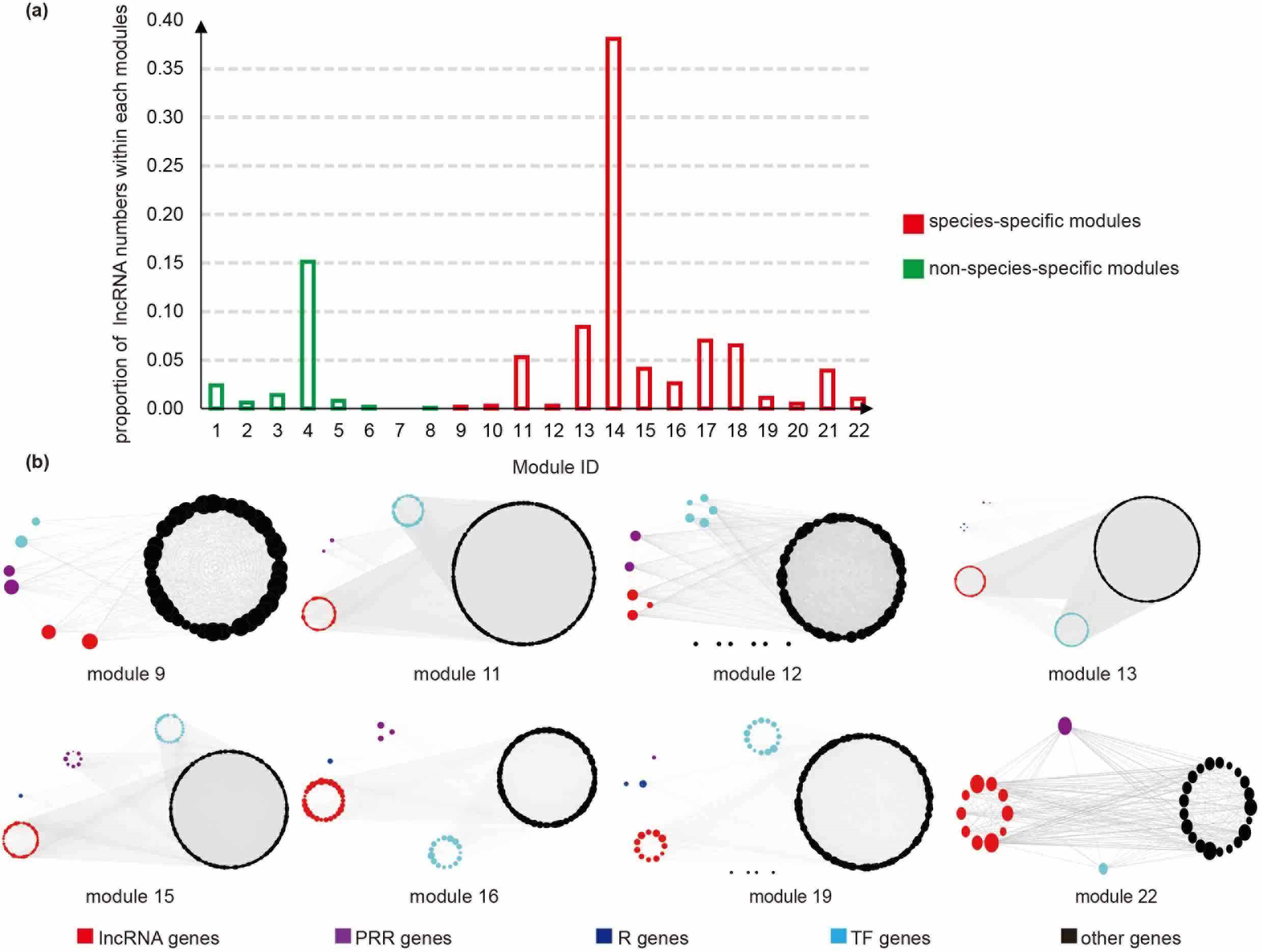

Supplementary Figure S11 Distribution of lncRNA transcripts in each module identified and network visualization of significant species-related modules. (a) Distribution of lncRNAs in each module. (b) Network visualization of significant species-related modules.

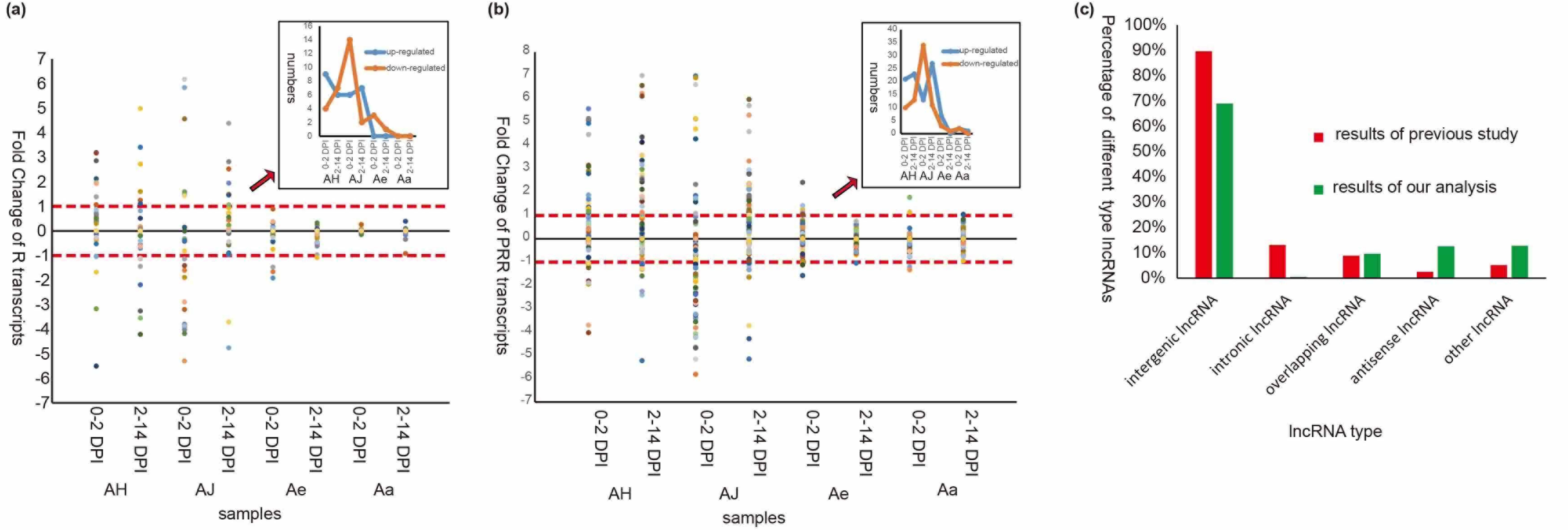

Supplementary Figure S12 Fold changes of R gene and PRR gene expression in four samples during Psa infection. (a) Fold changes of R gene expression. Each dot represents one R gene transcript. The upper panel illustrates the statistics of significantly differentially-expressed R gene transcripts in different samples and at different stages. (b) Fold changes of PRR gene expression. (c) Comparison of numbers of the different types of lncRNAs between the results reported by Tang et al. (Tang et al., 2016) and those obtained in our study.
